# *Bacillus subtilis* EpsA-O: A novel exopolysaccharide structure acting as an efficient adhesive in biofilms

**DOI:** 10.1038/s41522-024-00555-z

**Published:** 2024-10-02

**Authors:** Iztok Dogsa, Barbara Bellich, Mojca Blaznik, Cristina Lagatolla, Neil Ravenscroft, Roberto Rizzo, David Stopar, Paola Cescutti

**Affiliations:** 1 University of Ljubljana, Biotechnical Faculty, Department of Microbiology, Ljubljana, Slovenia; 2 Department of Advanced Translational Diagnostics, Institute for Maternal and Child Health, IRCCS “Burlo Garofolo”, Trieste, Italy; 3 University of Trieste, Department of Life Sciences, Via L. Giorgieri 1, Trieste, Italy; 4 University of Cape Town, Department of Chemistry, Rondebosch, South Africa

**Keywords:** Biofilms, Biological techniques

## Abstract

Extracellular polysaccharides are crucial components for biofilm development. Although *Bacillus subtilis* is one of the most characterized Gram-positive biofilm model system, the structure-function of its exopolysaccharide, EpsA-O, remains to be elucidated. By combining chemical analysis, NMR spectroscopy, rheology, and molecular modeling, high-resolution data of EpsA-O structure from atom to supramolecular scale was obtained. The repeating unit is composed of the trisaccharide backbone [→3)-β-d-Qui*p*NAc4NAc-(1→3)-β-d-Gal*p*NAc-(1→3)-α-d-Glc*p*NAc-(1]_n_, and the side chain β-d-Gal*p*(3,4-*S*-Pyr)-(1→6)-β-d-Gal*p*(3,4-*S*-Pyr)-(1→6)-α-d-Gal*p*-(1→ linked to C4 of GalNAc. Close agreement between the primary structure and rheological behavior allowed us to model EpsA-O macromolecular and supramolecular solution structure, which can span the intercellular space forming a gel that leads to a complex 3D biofilm network as corroborated by a mutant strain with impaired ability to produce EpsA-O. This is a comprehensive structure-function investigation of the essential biofilm adhesive exopolysaccharide that will serve as a useful guide for future studies in biofilm architecture formation.

## Introduction

*Bacillus subtilis* is one of the best studied Gram-positive model organisms. It is an important industrial organism, being proficient at secreting proteins and making small fine chemicals, as well as acting as a plant growth promoter^1^. It is ubiquitous, naturally transformable and has an extremely powerful genetic toolbox^2^. Its huge evolutionary and environmental success is supported by its ability to form biofilms on a variety of surfaces^3,4^. It is often used as a model system to dissect the mechanisms controlling biofilm extracellular matrix production and the subsequent transition from planktonic to sessile cells, biofilm development, differentiation, physiological heterogeneity, binary switching, quorum sensing, kin discrimination and dispersion^5–10^.

Great advances have been made in recent years in the characterization of *B. subtilis* biofilm matrix components, which include the secreted proteins TasA, TapA and BslA, as well as eDNA. Crystal structures of TasA (PDB ID: 5OF1) and BslA (PDB ID: 4BHU) have been resolved^11,12^. TapA acts as specific chaperone in TasA filament formation^13^, while recently the role of eDNA in early aggregate formation was demonstrated^14^. While previous research^15–21^ has revealed that the function of EpsA-O in *B. subtilis* extends beyond providing structural support to the biofilm (i.e. not only supports wrinkled colony morphology, but also plant root colonization, binding of metals, enables the osmotic spreading and protection from reactive oxygen species), its specific role in the microarchitecture of the biofilm and its chemical structure remains poorly understood.

**Fig. 1:**
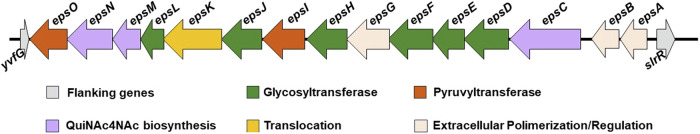
Structure of the *eps* locus of *B. subtilis subsp. subtilis* 6051-HGW. (portion 3514095-3529835 of accession number NC_020507.1). Arrows indicate the direction, relative length, and function (colored as per legend) of protein-coding genes.

EpsA-O is the main extracellular polysaccharide in *B. subtilis* and is synthesized by a cluster of 15 gene products of the *epsA–O* operon^15,22^ (Fig. 1). EpsA-O is required for complex colony structure and pellicle formation^15,23–25^. The function of only some gene products of the *epsA-O* cluster has already been demonstrated or predicted: EpsA and EpsB are tyrosine kinases that regulate Eps production; EpsC, EpsM and EpsN are responsible for the biosynthesis of *N*,*N*′-diacetylbacillosamine (QuiNAc4NAc), a modified monosaccharide synthesized by few bacteria^1^; EpsK is a membrane transporter similar to the Wzx protein of *E. coli*^26^; EpsL binds UDP-QuiNAc4NAc to the lipid acceptor undecaprenol phosphate (UndP) while the glycosyltransferase (GT) EpsD catalyzes the subsequent addition of the N-acetylglucosamine^27^.

In spite of several decades of active research, the exact composition of the repeating unit (RU) is still debated with findings from different studies in sharp disagreement^26,28,29^. A recent comprehensive revision^1^ concluded that the structural determination of EpsA-O is mandatory in order to move forward in the understanding of *Bacillus* *subtilis* biofilm architecture and function.

In this work the RU structure of EpsA-O was determined by chemical analysis, and 1D, and 2D NMR spectroscopy. Furthermore, the EpsA-O was characterized rheologically. The data were used to develop a string of beads model of the branched RU to simulate the volume space of the polysaccharide in solution. The results obtained were used to reevaluate the role of EpsA-O in biofilm function and development.

## Results

### EpsA-O has a decisive role in microstructure formation of the biofilm

**Fig. 2:**
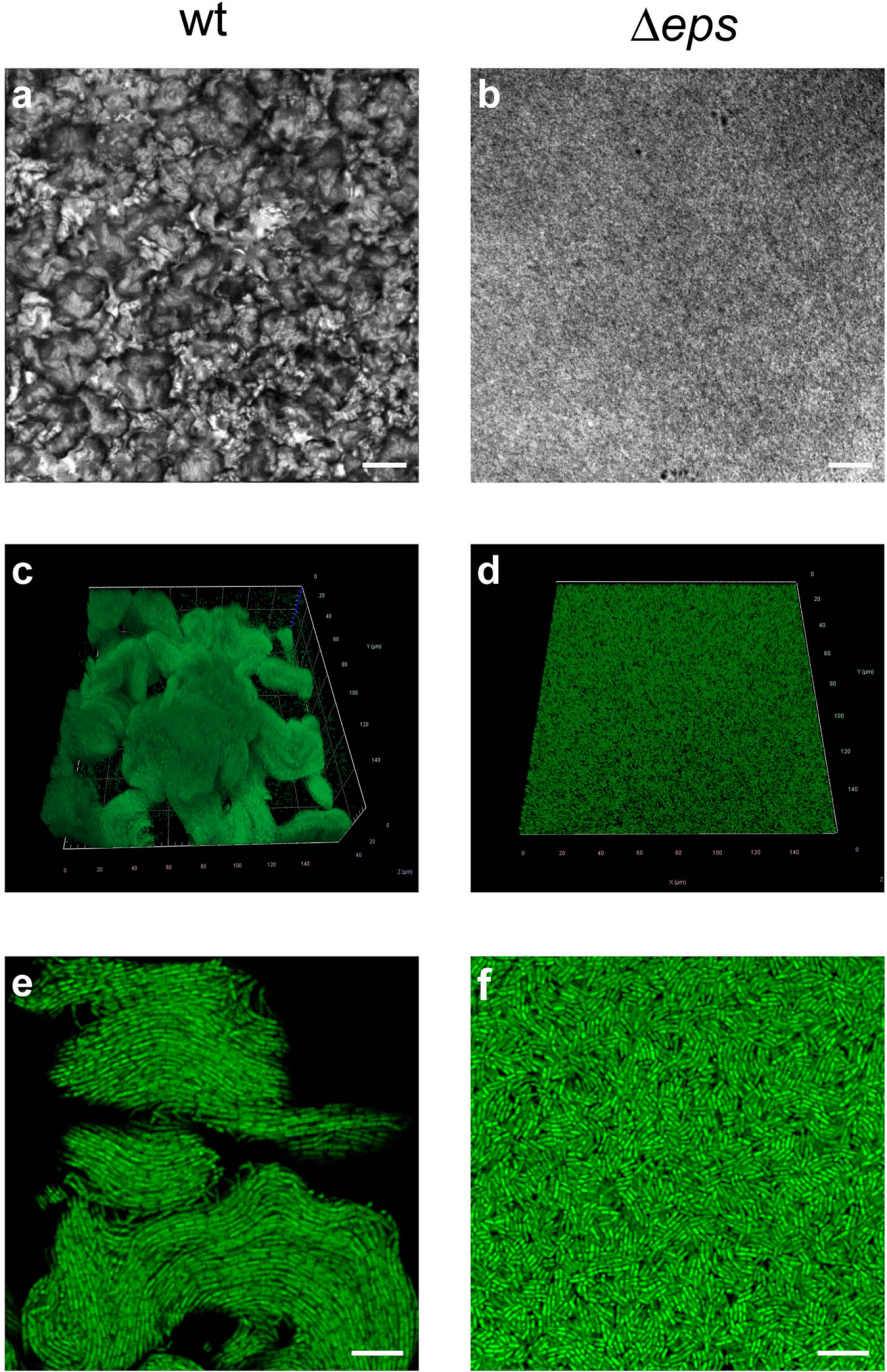
Microscopy images of *B. subtilis* biofilms. The mature biofilms were grown on agar-filled 3D-printed chamber slides and observed in situ. **a**, **b** Bright-field images taken by air objectives of WT and ∆*eps B. subtilis* biofilm surface, respectively. Scale bars represent 50 μm. **c** 3D-reconstructed CLSM image of upper 40 μm of WT biofilm. **d** 3D-reconstructed CLSM image of the upper 10 μm of ∆*eps* biofilm. **e**, **f** CLSM slice through the biofilm structure of WT and ∆*eps* biofilm, respectively. All CLSM images were taken by silicon oil objective. Scale bars represent 10 μm. To exclude pleiotropic effects as the main cause of the observed biofilm structural differences, further experiments were performed (Fig. 3).

**Fig. 3:**
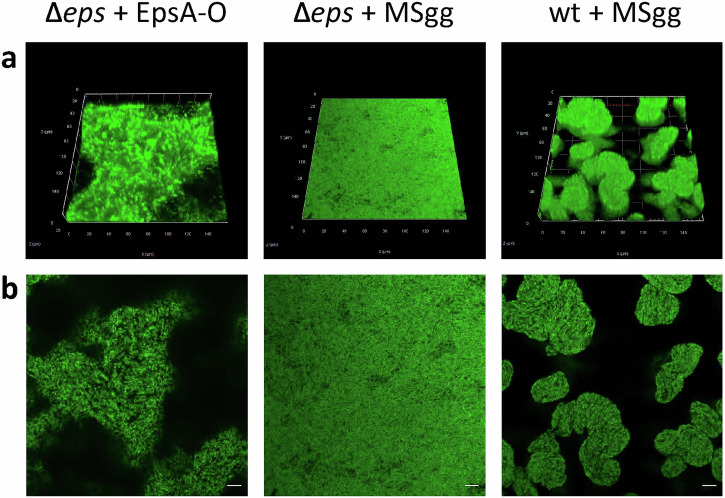
Microscopy images of native *B. subtilis* biofilms following the addition of exogenously purified EpsA-O or MSgg media. The addition of MSgg media or 0.2% exogenous EpsA-O occurred in three stages: at inoculation, and after 4 and 7 hours of incubation. Mature biofilms were cultivated on agar-filled 3D printed chamber slides and observed in situ. **a** A 3D-reconstructed CLSM image of mature biofilms. **b** CLSM slices through the biofilm structure of ∆*eps* and wt biofilms. All CLSM images were captured using a silicon oil objective, with scale bars indicating 10 μm.

In the past the CLSM microscopy has been successfully used to reveal the 3D microstructure of *B. subtilis* NDMed strain submerged native biofilms^30,31^. Here we report the 3D microstructure of native mature NCBI3610 *B. subtilis* biofilms on solid-air interface. The pronounced difference in topology in WT against *Δeps* biofilms was observed in bright-field microscopy (Fig. 2a, b), and in more details using confocal laser scanning microscopy (CLSM) (Fig. 2c, d). Upon 3D reconstruction, significant 3D structures were observed to form only in the WT biofilms. Optical sectioning of these biofilms (Fig. 2e, f) revealed that cells form compact directionally ordered fiber-like structures. When purified EpsA-O was exogenously added to the Δ*eps* mutant cells the roughness and patchiness substantially increased compared to the Δ*eps* mutant (Fig. 3). Compared to the wild type, however, roughness and local ordering were less pronounced. These images (Figs. 2, 3) suggest that EpsA-O acts as adhesive, structuring cells into 3D structures capable of resisting gravity. To resist gravity, the biofilm should be in a gel rather than sol state, which implies that the EpsA-O itself must be in a gel state in the biofilm. In order to elucidate the viscoelastic, physicochemical properties and structure of the EpsA-O, the polysaccharide was first isolated and purified in sufficient amounts.

### The purified EpsA-O has a high molecular mass

The EpsA-O raw isolate, obtained after optimization of the growth medium (Supplementary Fig. 1), was subjected to HPLC-SEC analysis; the elution profile (Supplementary Fig. 2a) showed two main peaks whereas the *Δeps* mutant profile did not contain the peak at *t* = 18 min. After performing additional steps of purification, (see Methods) the chromatogram of the purified EpsA-O displayed a single peak at *t* = 18 min (Supplementary Fig. 2b), containing <2% (w/w) of proteins and nucleic acids (Supplementary Fig. 2d–f). By applying the universal calibration method, the MM (molecular mass) of EpsA-O was estimated to be (2.5 ± 1) MDa. For such a large molecule, unusual high values of reducing sugar were obtained with the DNS test, even after extensive dialysis (Supplementary Fig. 2c). This was subsequently attributed to the high content of pyruvate substituent decorating EpsA-O, not the presence of carbonyl reducing ends.

### The EpsA-O forms gels, expands and displays polyelectrolyte properties

**Fig. 4:**
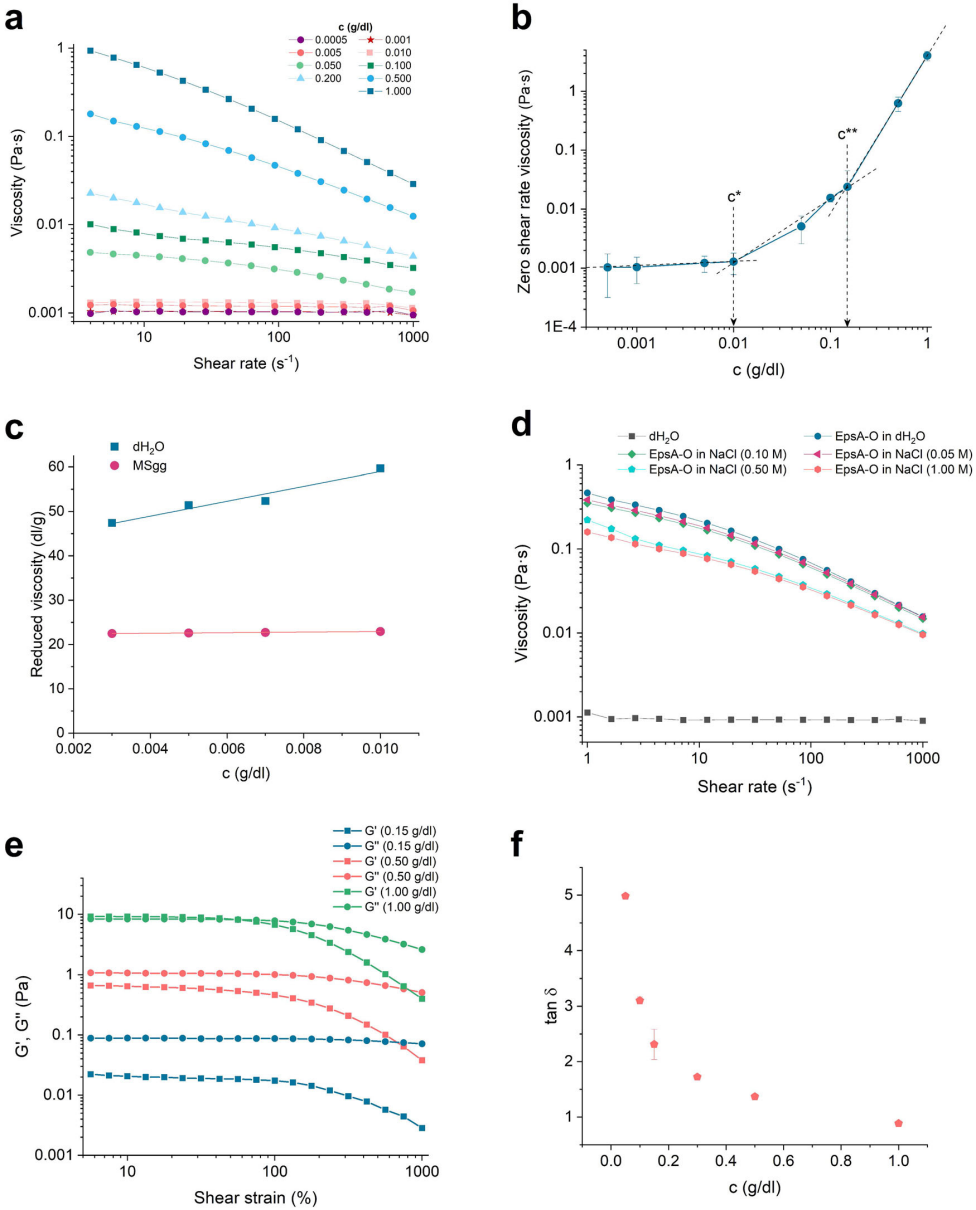
Rheology of EpsA-O. **a** The flow curves of EpsA-O dissolved in MSgg. The data points are color-coded and shaped according to the concentration of dissolved EpsA-O as follows: blue squares: 1 g/dL; blue circles: 0.5 g/dL; blue triangles: 0.2 g/dL; green squares: 0.1 g/dL; green circles: 0.05 g/dL; orange squares: 0.01 g/dL; orange circles: 0.005 g/dL; red stars: 0.001 g/dL; purple squares: 0.0005 g/dL. **b** Zero-shear viscosity of EpsA-O dissolved in MSgg at different concentrations indicating two critical concentrations, c* and c**. **c** Reduced viscosity (η_sp_/c) as a function of EpsA-O dissolved in dH_2_O or MSgg at different concentrations. Blue squares and trendline represent EpsA-O dissolved in dH_2_O, while red circles and trendline represent EpsA-O dissolved in MSgg. **d** Flow curves of EpsA-O aqueous solutions in the presence of increasing NaCl concentrations. Black squares: dH_2_O; blue circles: EpsA-O in dH_2_O (0.5 g/dL); red triangles: EpsA-O in 0.05 M NaCl (0.5 g/dL); green diamonds: EpsA-O in 0.1 M NaCl (0.5 g/dL); blue pentagons: EpsA-O in 0.5 M NaCl (0.5 g/dL); orange hexagons: EpsA-O in 1 M NaCl (0.5 g/dL). **e** Amplitude sweep test of EpsA-O dissolved in MSgg at different concentrations. The squares represent the storage modulus (Gʹ), and the circles represent the loss modulus (Gʺ). The color coding for the symbols and lines indicates different concentrations: green for 1 g/dL, orange for 0.5 g/dL, and blue for 0.15 g/dL. **f** Loss factor as a function of EpsA-O concentration in MSgg.

Purified EpsA-O solutions showed a pseudoplastic behavior suggesting inter-polymer interactions (Fig. 4a). The critical overlap concentration, c*, was at a value of (0.010 ± 0.005) g/dL (Fig. 4b). The semi-dilute regime ended with the second critical concentration, c** at (0.15 ± 0.05) g/dL that indicates physical entanglement of polymer chains above this concentration. The intrinsic viscosity, [η], which is a measure of the volume occupied by the polymer per unit weight (Fig. 4c), was (22.3 ± 0.1) dL/g in MSgg medium indicating that EpsA-O expands well in aqueous environment. The corresponding value in water was higher ([η] = (42 ± 2) dL/g) implying the polyelectrolyte nature of the polymer. This was further supported by conducting experiments with increasing NaCl concentrations (Fig. 4d). By increasing EpsA-O concentration, the viscoelastic liquid became a gel at concentrations ≥1.0 g/dL (Fig. 4e). The gel transition point did not change in the absence of salts (Supplementary Fig. 3a), suggesting that the major gelling mechanism does not involve ions. At 1% EpsA-O concentration the gel was weak: it yielded at 25% shear strain deformation and collapsed at 80% shear strain (Fig. 4e). The energy needed to irreversibly deform the structure has to exceed the cohesive energy, which was calculated to be 3 ×10^−28 ^J/nm^3^ for EpsA-O gel. The phase transition to the gel state is a second-order continuous transition depending on EpsA-O concentration in solution (Fig. 4f).

When purified EpsA-O was exogenously added to Δ*eps* mutant cells, the rheological behavior changed significantly (Supplementary Fig. 3b). Both storage and loss modulus increased by an order of magnitude. More importantly, the qualitative behavior of the material changed as well. The loose cell material of Δ*eps* mutant that behaved as a weak viscoelastic liquid material changed to viscoelastic solid material. This is indicated by a decrease in loss factor (tan δ = G”/G’) which changed from 1.3 to 0.3 upon EpsA-O addition. This is a strong indication that *B. subtilis* cells in the presence of EpsA-O form a gel material.

### Composition and linkage analysis of the exopolysaccharide EpsA-O produced by *B. subtilis*

Acid hydrolysis of EpsA-O followed by derivatization to alditol acetates gave the neutral sugars Gal, GlcNAc, GalNAc and Glc in the molar ratios 7.7:1.1:1.0:0.6. Methanolysis followed by derivatization to trimethylsilylethers showed Gal, GlcNAc and GalNAc in the molar ratios 2.2:1.5:1.0. Glycosidic linkage analysis identified 6-Gal*p*, 3,4-Gal*p*, 3,4,6-Gal*p*, 3-Glc*p*NAc, and 3,4-Gal*p*NAc in the molar ratios 1.0:1.0:0.6:1.2:1.1. A very small late eluting peak with key primary fragments at 159 and 172 *m/z*, and secondary fragments at 117 and 130 *m/z* was attributed to 3-Qui*p*NAc4NAc. The presence of three branched sugars and the absence of terminal non-reducing residues suggested heavy substitution by non-carbohydrate moieties. The hexoses all have the d absolute configuration.

### ^1^H NMR spectroscopy of EpsA-O produced by *B. subtilis*

**Fig. 5:**
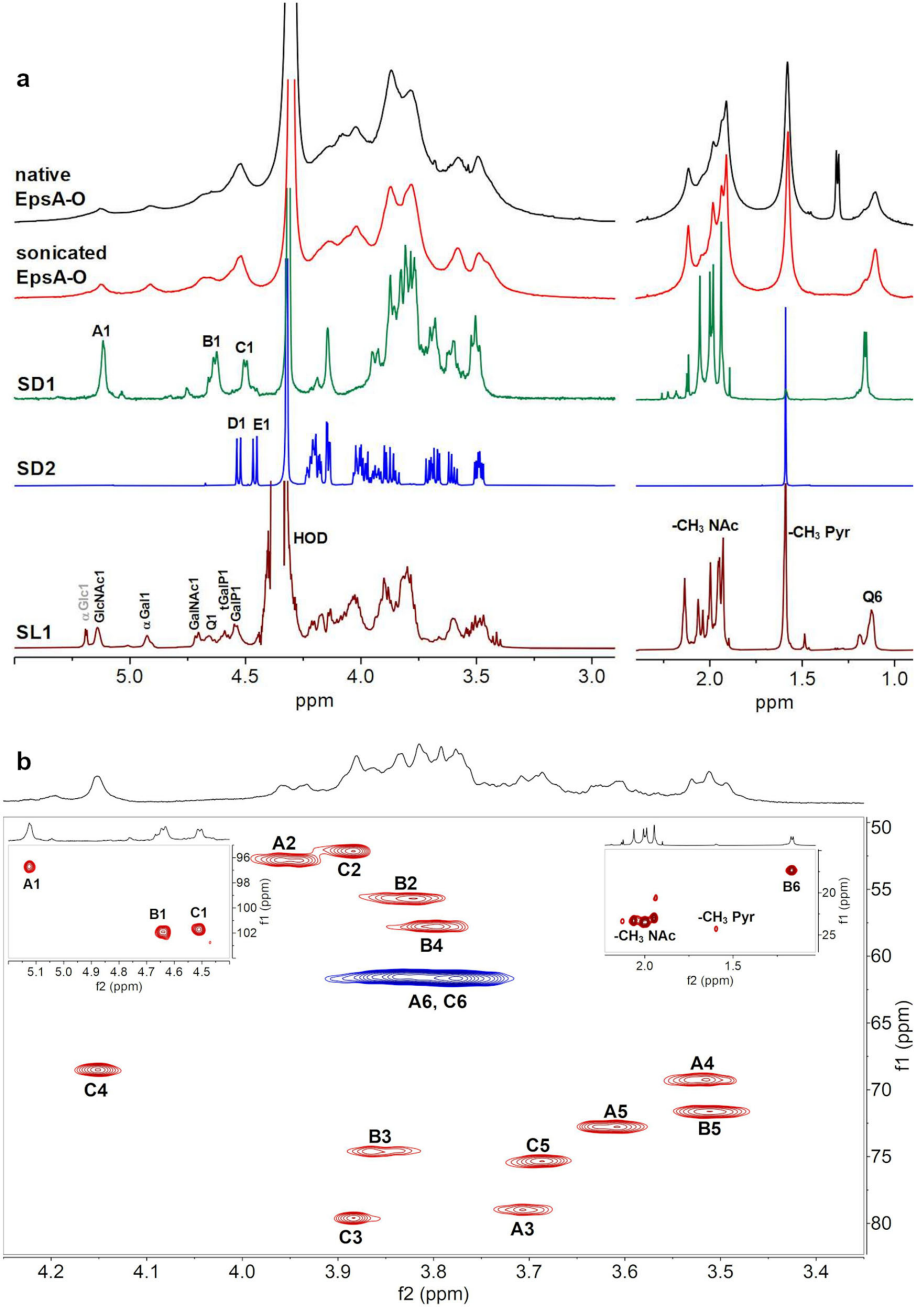
1D and 2D NMR spectroscopy data of EpsA-O, and SD1 and SD2 samples obtained by Smith degradation. **a** ^1^H NMR spectra of native EpsA-O, sonicated EpsA-O, **SD1,** **SD2**, and EpsA-O after solvolysis recorded at 70 °C. Anomeric resonances are labelled with letters and sugar abbreviations, as in Tables 1–3. **b** Ring region of the **SD1** HSQC spectrum recorded at 70 °C. Anomeric and methyl regions are reported in the insets. Cross peaks are labeled as in Table 1 (**A**1 = H1/C1 of residue **A**).

The ^1^H NMR spectrum of the native EpsA-O showed extremely broad lines (Fig. 5a) which did not become sharper upon sonication. Therefore, integration of the methyl region of the sonicated sample gave an approximate estimation of 1 deoxy-sugar (at 1.1 ppm), ∼1.8 pyruvyl ketal substituents (at 1.6 ppm) and ∼4 N-acetyl groups near 2 ppm. Chemical analysis detected only two N-acetylated sugars (GlcNAc and GalNAc), thus NMR data suggested the presence of a 6-deoxy doubly N-acetylated residue.

### Smith degradation of EpsA-O produced the polysaccharide backbone and part of the side chain

Since the 6-linked Gal is the only residue susceptible to periodate oxidation, the EpsA-O was subjected to complete Smith degradation^32^ to yield three fractions, **SD1,** **SD2** and **SD3**, isolated by size-exclusion chromatography (Supplementary Fig. 4a).

The ^1^H-NMR spectrum of **SD1** (Fig. 5a) showed three anomeric signals of similar intensity at 5.12 (**A**), 4.64 (**B**), and 4.51 (**C**) ppm, indicative of one alpha and two beta anomeric residues. These data together with the absence of reducing end resonances (see inset HSQC plot, Fig. 5b) showed that **SD1** is a polymer composed of a trisaccharide repeating unit (RU). Diagnostic signals in the methyl region were assigned to H6 of a deoxy residue (1.17 ppm, 3 protons), and four N-acetyl substituents (1.95, 1.99, 2.00, and 2.06 ppm, 12 protons). A small signal at 1.59 ppm (0.36 protons) indicated a small amount of pyruvate in comparison with the native EpsA-O (6 protons) and confirmed the success of the Smith degradation.

The structure of **SD1** RU was determined by use of an array of ^1^H-^1^H homonuclear experiments (COSY, TOCSY (Supplementary Fig. 5), NOESY, and ^1^H-^13^C heteronuclear correlation experiment (HSQC). In the HSQC spectrum (Fig. 5b) the proton/carbon pairs are labeled and the NMR data are collected in Table 1. Finally, the sequence of sugar residues followed from the NOESY inter-residue correlations, as shown in Supplementary Fig. 6. NMR data agree with those reported for *Bacillus licheniformis* ATCC 9945 exopolysaccharide^33^. The magnitude of the glycosylation shift of β-d-Gal*p*NAc C3 (+7.6 ppm) is identical to the reported^33^, thus establishing the absolute configuration of β-Qui*p*NAc4NAc to be d. Therefore, NMR analysis established the structure of the backbone RU of EpsA-O (structure 1).

$${\left[\rightarrow\!\! 3\right){\mbox{-}}\upbeta{\mbox{-}} {\scriptstyle{\rm{D}}}{\mbox{-}{\mathrm{QuipNAc4NAc}}{\mbox{-}}}(1\!\! \rightarrow\!\! 3){\mbox{-}}\upbeta{\mbox{-}} {\scriptstyle{\rm{D}}}{\mbox{-}}{\mathrm{Gal}}{p}{\mathrm{NAc}}{\mbox{-}}(1\!\!\rightarrow\!\! 3){\mbox{-}}{\upalpha{\mbox{-}}} {\scriptstyle{\rm{D}}}{\mbox{-}}{\mathrm{GlcpNAc}}{\mbox{-}}\left(1\right]_n}$$

structure 1: backbone repeating unit of EpsA-O

**Table 1:** ^1^H and ^13^C chemical shift assignments of the sample **SD1** recorded in D_2_O at 500 MHz and 70 °C

Residue	Nucleus	Chemical shifts (ppm) ^a^					
		1	2	3	4	5	6
**A**	^1^H	5.12	3.95	3.70	3.51	3.61	3.83 - 3.76
**→3)-α-d-Glc*p*NAc-(1→**	^13^C	96.7	52.8	79.0	69.3	72.8	61.7
**B**	^1^H	4.64	3.83	3.85	3.80	3.50	1.17
**→3)-β-Qui*p*NAc4NAc-(1→**	^13^C	101.9	55.7	74.6	57.8	71.6	17.3
**C**	^1^H	4.51	3.88	3.88	4.15	3.69	3.83 - 3.76
**→3)-β-d-Gal*p*NAc-(1→**	^13^C	101.7	52.1	79.6	68.6	75.3	61.7

^a^ Chemical shifts are given relative to internal acetone (2.225 ppm for ^1^H and 31.07 ppm for ^13^C).

The ^1^H-NMR spectrum of **SD2** (Fig. 5a) showed two beta anomeric signals at 4.54 (**D**) and 4.47 (**E**) ppm (J _H1-H2_ = 8.2 Hz), and a methyl signal at 1.60 ppm (6 protons) assigned to pyruvate. As for **SD1**, the use of COSY, TOCSY (Supplementary Fig. 7), and HSQC (Supplementary Fig. 8) led to the assignments of all chemical shifts, reported in Table 2, in agreement with literature values^33,34^, establishing that **SD2** has the following structure (structure 2):

$${\upbeta{\mbox{-}}{\scriptstyle{\rm{D}}}{\mbox{-}}{\mathrm{Gal}}{p}{(3,\!4{\mbox{-}}}{\mathrm{S}}{\mbox{-}}{\mathrm{Pyr}}){\mbox{-}}(1\!\! \rightarrow \!\!6){\mbox{-}} \upbeta {\mbox{-}}{\scriptstyle{\rm{D}}}{\mbox{-}}{\mathrm{Gal}}{p}{(3,\!4{\mbox{-}}{\mathrm{S}}{\mbox{-}}{\mathrm{Pyr}}){\mbox{-}}\left(1\!\! \rightarrow \!\! {\mathrm{Gro}}\right.}}$$

structure 2: oligosaccharide **SD2**

**Table 2:** ^1^H and ^13^C chemical shift assignments of the sample **SD2** recorded in D_2_O at 500 MHz and 70 °C

Residue	Nucleus	Chemical shifts (ppm) ^a^					
		1	2	3	4	5	6
**D**	^1^H	4.53	3.49	4.20	4.15	4.02	3.90
β-d-Gal*p*-3,4 Pyr-(1**→**	^13^C	103.3	74.2	79.5	75.6	74.0	61.7
**E**	^1^H	4.46	3.49	4.20	4.15	4.22	4.19 – 4.02
**→**6)-β-d-Gal*p* 3,4 Pyr(1**→**	^13^C	103.1	74.2	79.5	75.6	73.0	69.8
Pyr S	^1^H			1.60			
	^13^C	177.9	108.7	24.3			
		H1’a	H1’b	H2	H3’a	H3’b	
1-Gro	^1^H	3.99	3.70	3.94	3.68	3.60	
	^13^C	72.0	72.0	71.6	63.4	63.4	

^a^Chemical shifts are given relative to internal acetone (2.225 ppm for ^1^H and 31.07 ppm for ^13^C).

Glycerol (Gro) linked through C1 derives from a 6-linked hexose upon Smith degradation^35^, thus indicating that the pyruvylated disaccharide is linked to C6 of Gal in the native exopolysaccharide, in agreement with the linkage analysis. A 1D NOESY experiment established the S configuration of the pyruvyl substituents^36^.

Moreover, upon elucidation of the ^1^H-NMR spectrum (Supplementary Fig. 4b) the structure of **SD3** was established as follows (structure 3):

$${\upbeta{\mbox{-}} {\scriptstyle{\rm{D}}}{\mbox{-}}{\mathrm{Gal}}}{p}{(3,\!4{\mbox{-}}{\mathrm{S}}{\mbox{-}}{\mathrm{Pyr}}){\mbox{-}}\left(1 \!\! \rightarrow\!\!{\mathrm{Gro}}\right.}$$

structure 3: oligosaccharide **SD3**

### NMR spectroscopy of fraction SL1 obtained upon solvolysis of EpsA-O

In order to obtain NMR spectra of the EpsA-O containing information on the intact repeating unit, including the 6-Gal residue, the MM of the polysaccharide was decreased by treatment in concentrated TFA (solvolysis) to yield the fraction named **SL1** (Supplementary Fig. 9). The ^1^H NMR spectrum (Fig. 5a) anomeric region showed three separated signals at 5.19, 5.14 and 4.92 ppm (α-sugars), and two clusters of overlapping resonances at 4.75-4.45 ppm (β-sugars). The methyl region contained the expected signals for H6 of QuiNAc4NAc, N-acetyl groups and pyruvyl substituents with the presence of more than four NAc and two H6 signals indicated some sample heterogeneity arising from solvolysis. Comparison of **SL1** ^1^H NMR spectrum with those of **SD1** and **SD2** identified the resonance at 5.14 ppm as H1 of α-d-GlcNAc and the signal at 4.92 ppm as H1 of 6-linked Gal, present in the NMR of the sonicated EpsA-O (Fig. 5a) but not of the Smith degradation products. The signal at 5.19 ppm was assigned to H1 of a glucose belonging to another polymer, as it is very sharp compared to all EpsA-O resonances, it was not consistently present in all EpsA-O batches and size-exclusion chromatography yielded fractions with variable content. Moreover, the same α-Glc containing polymer was the only carbohydrate produced by a Δ*eps* mutant strain (Supplementary Fig. 10), indicating the presence of a yet unknown exopolysaccharide in *B. subtilis*.

**Fig. 6:**
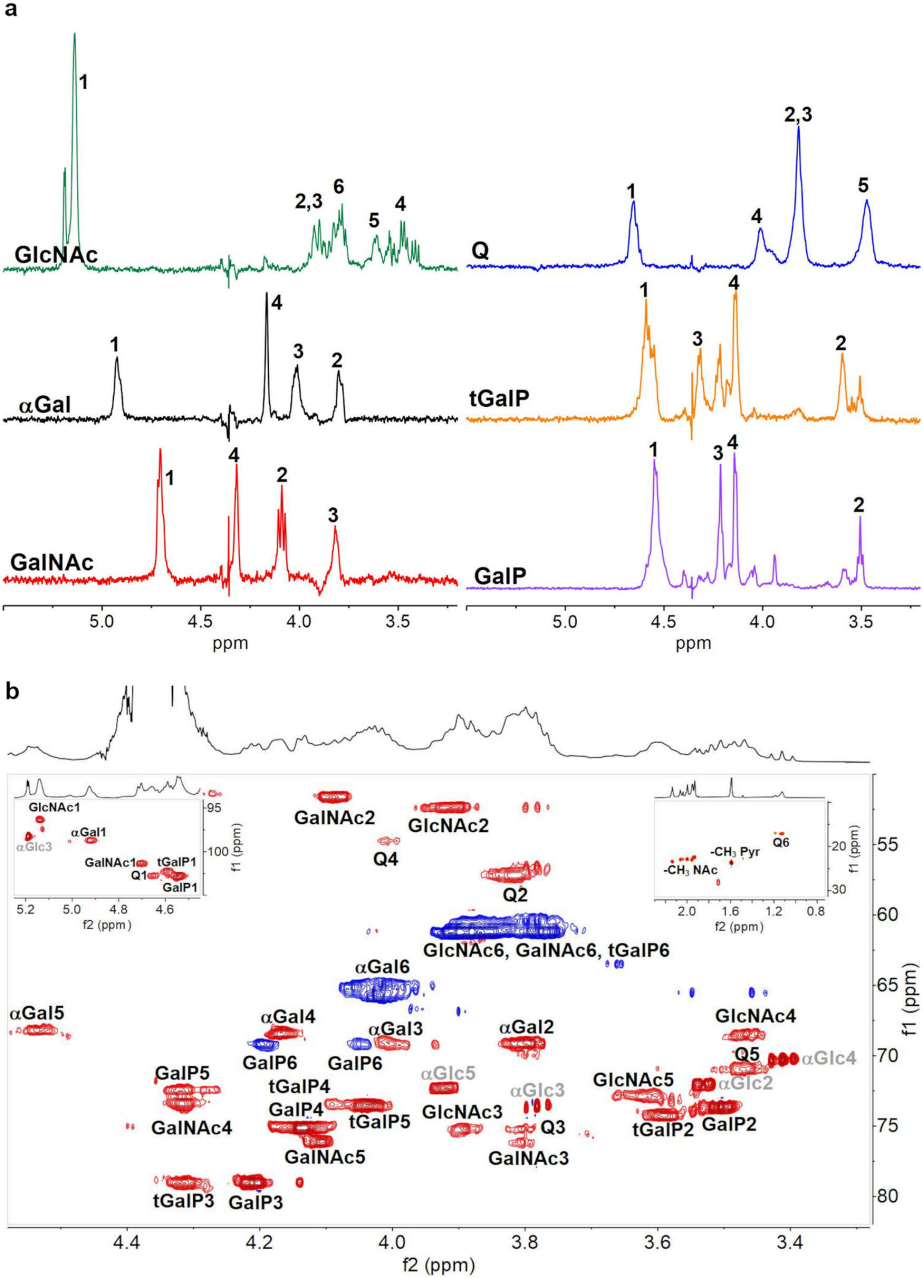
1D and 2D NMR spectroscopy data of EpsA-O after solvolysis. **a** 1D TOCSY spectra extracted from 2D spectra of sample **SL1** obtained after solvolysis of EpsA-O recorded at 70 °C. **b** Ring region of the HSQC spectrum recorded at 70 °C of the sample **SL1**. Anomeric and methyl regions are reported in the insets. Peaks and cross peaks are labeled as in Table 3. Signals belonging to a glucose residue not part of the EpsA-O are labeled in grey.

The structure of **SL1** RU was determined by use of COSY, 1D-TOCSY (Fig. 6a), NOESY, and HSQC (Fig. 6b) experiments. Starting from the proton assignments (Fig. 5a) and by comparison of the **SL1** HSQC with those of **SD1** and **SD2**, the chemical shifts for each spin system were established (Table 3) and the values agree with literature data^33,37^. Finally, 1D NOESY of the 6-Gal H1 showed its binding to GalNAc C4 (Supplementary Fig. 11), thus establishing the following structure of the EpsA-O RU (structure 4, Supplementary Fig. 12):



structure 4: main repeating unit of EpsA-O

**Table 3:** ^1^H and ^13^C chemical shift assignments of the sample **SL1** obtained upon solvolysis of EpsA-O recorded in D_2_O at 600 MHz and 70 °C

Residue	Nucleus	Chemical shifts (ppm) ^a^					
		1	2	3	4	5	6
**→3)-α-d-Glc*p*NAc-(1→**	^1^H	5.14	3.92	3.90	3.47	3.61	3.77-390
**(GlcNAc)**	^13^C	96.2	52.4	75.3	68.6	73.0	61.2
**→6)-α-d-Gal*p*-(1→**	^1^H	4.92	3.80	4.00	4.17	4.53	4.01
**(αGal)**	^13^C	98.8	69.2	69.2	68.5	68.3	65.2
**→3,4)-β-d-Gal*p*NAc-(1→**	^1^H	4.70	4.09	3.82	4.32	4.10	3.77-390
**(GalNAc)**	^13^C	101.4	51.7	76.3	73.5^b^ (72.6)^b^	76.1	61.2
**→3)-β-d-Qui*p*NAc4NAc-(1→**	^1^H	4.65	3.82	3.81	4.00	3.47	1.13
**(Q)**	^13^C	102.8	57.2	75.3	54.8	71.1	17.1
**β-d-Gal*p*(3,4-S-Pyr)-(1→**	^1^H	4.59	3.60	4.32	4.15	4.03	3.77-390
**(tGalP)**	^13^C	102.3	74.4	79.1	75.3	73.5	61.2
**→6)-β-d-Gal*p*(3,4-S-Pyr)-(1→**	^1^H	4.54	3.51	4.21	4.15	4.32	4.19 – 4.05
**(GalP)**	^13^C	102.8	73.8	79.1	75.3	72.6^b^ (73.5)^b^	69.2

^a^Chemical shifts are given relative to internal acetone (2.225 ppm for ^1^H and 31.07 ppm for ^13^C).

^b^Signals may be exchanged.

Moreover, the 2:1 ratio of **SD2**/**SD3** obtained from the size exclusion chromatogram together with the 3,4,6-Gal*p* ratio of 0.6 to 1.0 6-Gal*p* suggests side chain variability with up to one-third of the repeating units having a disaccharide with only one pyruvylated Gal instead of trisaccharide side chain. This is in agreement with a pyruvylation degree of 0.13 ± 0.02 estimated by chemical analysis. Since EpsA-O was purified by repeated treatment with 0.1 M NaOH, the possible presence of alkali labile groups such as acetates could not be established.

### Prediction of the reactions catalysed by the EpsA-O gene cluster proteins

Knowledge of the EpsA-O RU structure aided for assignment of the functions of the previously uncharacterized genes *epsE*, *epsF, epsG, epsH, epsI, epsJ*, and *epsO* involved in the EpsA-O biosynthesis. This was achieved by a bioinformatic analysis searching for conserved domains (CD) in the proteins encoded by the *eps*A-O cluster. Identified CDs were queried in the CD database (CDD) to find annotated proteins with an enzymatic function consistent with the reactions required for RU biosynthesis. In the case of glycosyltransferases, the CAZY database (http://www.cazy.org/) was consulted for a more detailed understanding of the retaining or inverting mechanism of action. The results obtained and a detailed explanation of the bioinformatic work performed are reported in the Supplementary file (Supplementary Table 1, Supplementary Figs. 13-15).

**Fig. 7:**
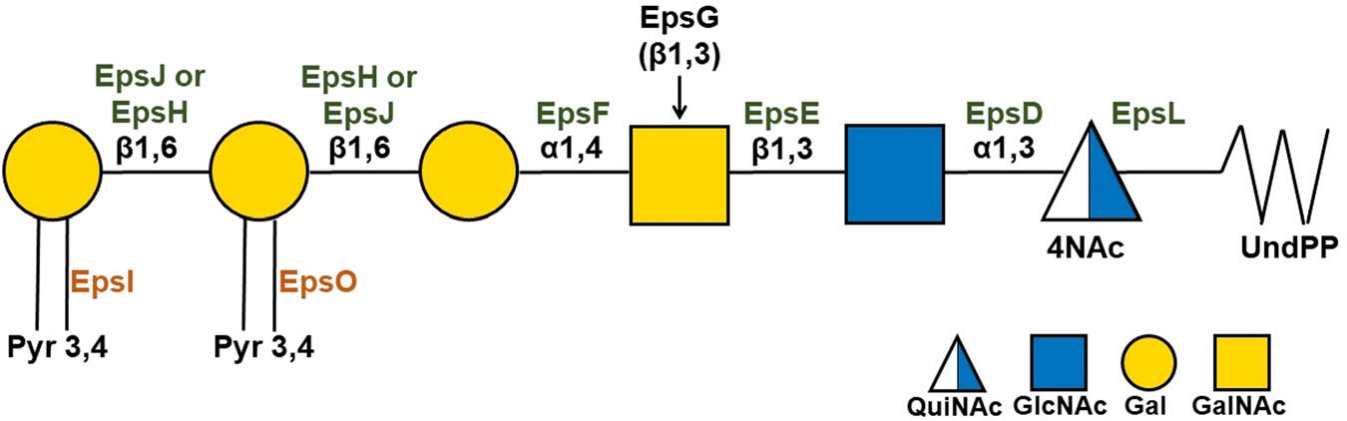
*eps*A-O gene functions. Proposed glycosyltransferase, pyruvyltransferase, and polymerase activity of the proteins encoded by the *eps* gene cluster in the biosynthesis of the EpsA-O repeating unit.

During the biosynthesis of the EpsA-O RU, ketal-pyruvylation of both β-galactose residues occurs before polymerization. Although pyruvylation happens in many glycan structures from bacteria to yeasts, few studies investigated the enzymes governing these reactions. All agree that the reaction occurs in the cytoplasm with the lipid-PP-linked RU as acceptor, but the time at which pyruvylation occurs may vary, depending on the position of the pyruvylated residue in the RU^38^. The annotation of EpsI and EpsO as putative pyruvyltransferases is strongly supported by the presence of three extremely conserved amino acids, suggested as fundamental for *Saccharomyces pombe* pyruvyltransferase activity^39^ (Supplementary Fig. 15). The sequence of EpsI and EpsO was compared with that of WcuL^40^ which is a pyruvyltransferase catalyzing the addition of a pyruvate group on a terminal β-d-Gal in *Klebsiella pneumoniae* K2 and K30, thus forming 3,4-Pyr-β-d-Gal. Since BLAST alignment showed that WcuL has higher homology with EpsO than with EpsI (Table S1), pyruvylation of the first β-d-Gal which leads to a terminal 3,4-Pyr-β-d-Gal, a sub-structure identical to that of polysaccharides K2 and K30, is likely catalyzed by EpsO. Then the second β-d-Gal is added to the 3,4-Pyr-β-d-Gal and pyruvylated by EpsI, whose lower similarity to WcuL is probably due to the different acceptor molecule recognized. The biological RU of EpsA-O and the assignments of the gene functions are reported in Fig. 7. The published data on *epsA*, *epsB, epsC, epsK, epsL, epsM* and *epsN* functions (Supplementary file) together with our in-silico genes functions predictions, describing six GTs and two pyruvyl transferases among other proteins, perfectly match the structure of EpsA-O.

### Modeling EpsA-O spatial structure reveals that EpsA-O is a carefully tailored biofilm adhesive

**Fig. 8:**
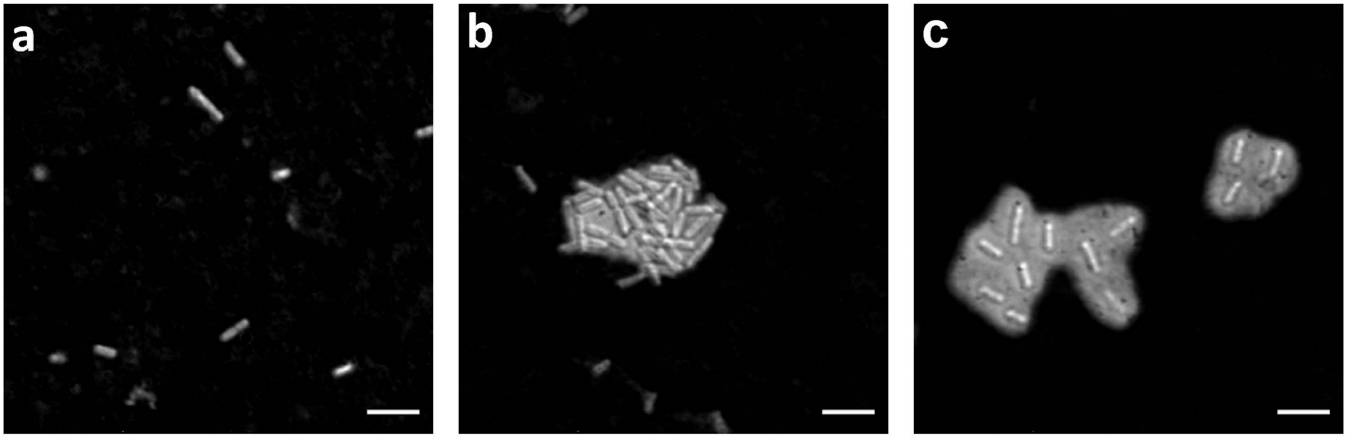
Negative staining with Indian ink of the *B. subtilis* wild-type and mutants. DIC microscopic images of *B. subtilis* Δ*eps* (**a**), wt (**b**), and Δ*sinR* strain (**c**), where polysaccharide capsules were clearly visible around the wild type, and Δ*sinR* strain. Scale bars represent 5 μm.

To visualize EpsA-O by CLSM in the intercellular volume several fluorescent dyes were applied. Concanavalin A (ConA) and wheat germ agglutinin (WGA) did not show specificity against EpsA-O, consistent with their ability to stain teichoic acids in the cell walls of *B. subtilis*^41–44^. As staining by Calcofluor-white produced only weak unspecific fluorescence, we switched to wide-field microscopy techniques. Indian ink stained *B. subtilis* cells, observed by DIC, confirmed the ability of EpsA-O to fill the intercellular space (Fig. 8) enabling cell-cell aggregation.

To visualize EpsA-O in 3D solution space and its ability to act as an adhesive at the molecular level, the EpsA-O in the intercellular volume space was investigated using the String-of-beads model^45–47^. The advantage of this modeling approach is that it can relate the primary structure of EpsA-O to the macromolecular descriptors such as MM and intrinsic viscosity to yield a statistical spatial 3D polymer structure in a given space.

**Fig. 9:**
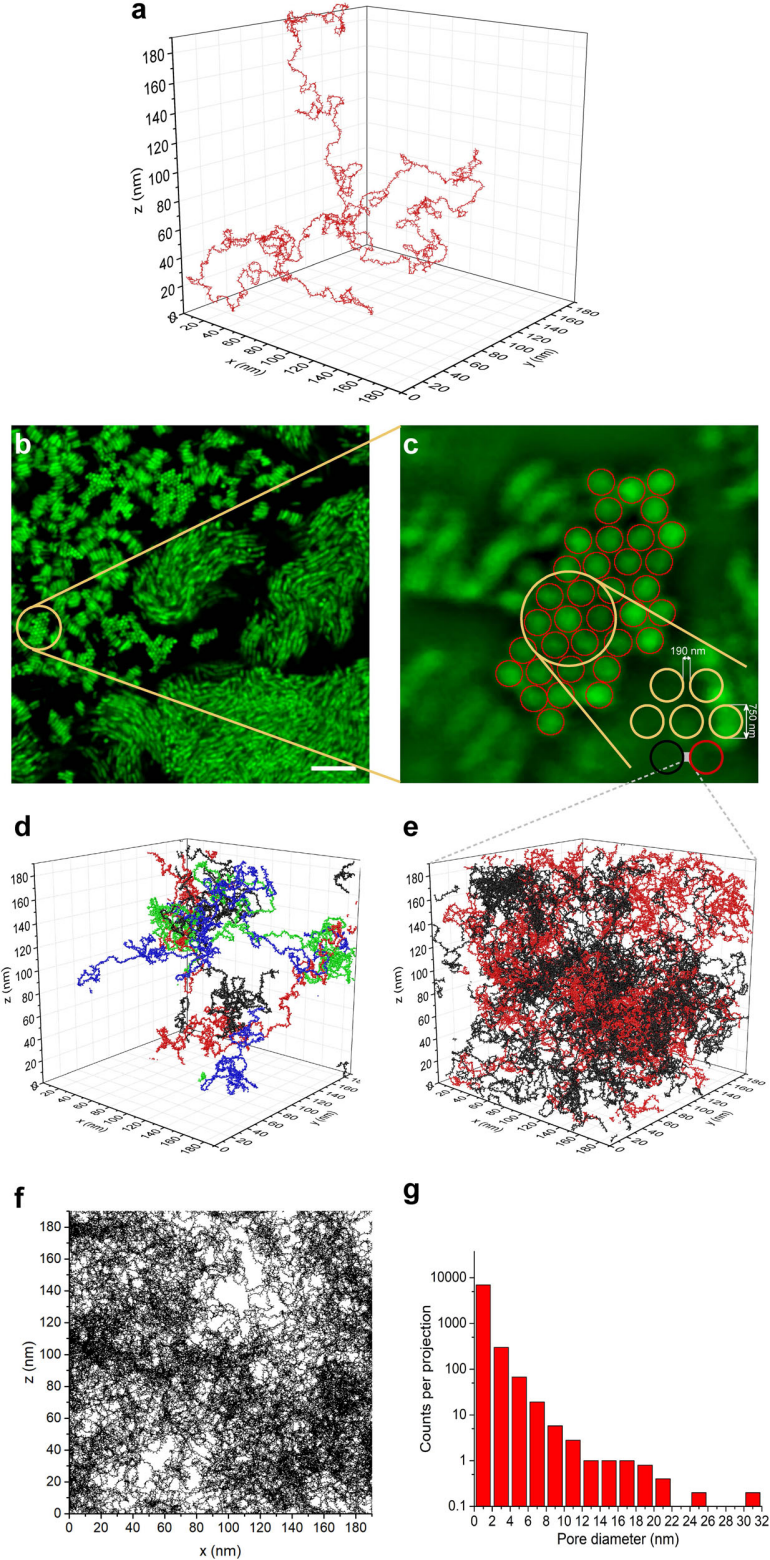
EpsA-O in the intercellular space of *B. subtilis* biofilm. **a** The volume space of a single EpsA-O molecule. By combining the data obtained by universal calibration method, intrinsic viscosity and NMR spectroscopy, the EpsA-O molecule was modeled with a String-of-beads model. The representative structure is a symmetrical comb with 0.5 degree of branching and 78 nm radius of gyration. The backbone has a persistence length of 11 nm. The polymer is composed of 12500 impenetrable beads of diameter 0.52 nm corresponding to the size of glucose molecule. **b** The mature WT biofilms were partially disrupted to obtain a better view into cell fibers composing the 3D structures. Scale bar represents 10 μm. **c** The volume fraction of intercellular space and the average distance between two neighboring cells were estimated to be (50 ± 10) % and 190 nm, respectively. **d** In the box of 190 × 190 × 190 nm^3^, EpsA-O was modeled at concentration slightly above c** (see Fig. 4b); each molecule is in different color. **e** The same box, but the opposing xz sides, representing the cell envelopes of two neighboring cells, were set to be impenetrable and were the starting points of growing EpsA-O molecules, 10 EpsA-O molecules for each bacterium (red for bacterium 1 and black for bacterium 2) are shown, corresponding to the actual EpsA-O concentration. **f** xz projection of the space in **e**, showing the empty space (pores) between bacterium 1 to bacterium 2. **g** Distribution of pore diameters as determined from five independent projections.

According to its primary structure (Structure 4), the EpsA-O degree of branching, (DB) is 0.5. Therefore, the compression factor for EpsA-O (*g*_*[η]*_) is 0.5, which means that the hypothetical non-branched variant of EpsA-O of identical MM would have twice the intrinsic viscosity of the branched variant (see Material and methods, Modeling spatial structure of EpsA-O). By using the Yamakawa model^48^, the persistence length, *lp*, of the non-branched EpsA-O was estimated to be 11 nm. Upon application of the String-of-beads model, the non-branched EpsA-O variant structure with the desired *lp* of 11 nm was obtained by setting a random torsion angle and limiting the bond angle to 0.6 rad. To simulate the branched polysaccharide, the same parameters were used after adding side chains on every 3^rd^ monosaccharide of the backbone, resulting in a rather large structure (Fig. 9a) that fits into a space of 200 × 200 × 200 nm^3^.

To check how such a structure fits into the *B. subtilis* biofilm space, the supramolecular structure of EpsA-O was simulated at EpsA-O concentrations measured in WT biofilm. The average experimental EpsA-O concentration was (0.61 ± 0.05) mg per 100 mg of wet biofilm which is significantly higher than c**, suggesting that single polymer chains may overlap in biofilm. Although this is below the gelling concentration (c ≥ 1.0 g/dL ≈ 1 mg per 100 mg) required for gelation of the biofilm, the local concentration of EpsA-O in the intercellular space can be significantly higher due to a large cell volume fraction in the biofilm (Fig. 2e). To get a better insight into intercellular volume, the native biofilm was partially disrupted giving rise to the formation of ordered cell clusters (Fig. 9b). Some of the clusters aligned along the z-axis (Fig. 9c), enabling accurate determination of the intercellular distance at 190 nm. Using 750 nm as the average diameter of *B. subtilis* cells^49^, the volume fraction of the extracellular space was estimated to be (50 ± 10) %, thus increasing the local concentration of EpsA-O to 1.2 g/dL, above its gelling point. By randomly positioning the origins of four polymer chains into the modeling box of 190 × 190 × 190 nm^3^, EpsA-O supramolecular structure at concentration slightly above c** (at 0.24 g /dL) was simulated (Fig. 9d) and chain entanglements were observed. To get closer to the native state, twenty EpsA-O chains, corresponding to the concentration of 1.2 g/dL, were inserted into the box from opposite sides, simulating EpsA-O production by neighboring cells in the biofilm. The polymer chains entangled across the box (Fig. 9e), thus providing a molecular explanation for the observed gel formation in the biofilm. To estimate the porosity of the resultant entangled network of EpsA-O, 2D projections of x-z plane (Fig. 9f) of the simulated space were constructed and a distribution of different size pores was observed. Although the majority of the pores had diameters < 2 nm (Fig. 9g), there were several of about 10 nm which allow diffusion of polymers within the extracellular space.

## Discussion

The present investigation resolves the structure and function of EpsA-O in *B. subtilis* biofilms. For the first time combined chemical and NMR spectroscopy analyses were used to determine the fine structure of the EpsA-O extracted from pellicles. The primary structure of the main EpsA-O RU consists of a novel branched hexasaccharide made of a trisaccharidic backbone, identical to that of the exopolysaccharide produced by *B. licheniformis*^33^, and a trisaccharide side chain decorated with two pyruvyl substituents (structure 4). The minor contribution of a disaccharide side chain lacking one pyruvylated Gal was also detected. This makes EpsA-O a novel and unique structure among microbial polysaccharides. Attribution of the glycosyltransferases to specific catalyzed reactions was achieved by in silico analysis with a good degree of homology. However, the uniqueness of the side chain structure, β-d-Gal*p*(3,4-*S*-Pyr)-(1→6)-β-d-Gal*p*(3,4-*S*-Pyr)-(1→6)-α-d-Gal*p*-(1→, impeded to find suitable GTs for sequence homology analysis in data bases, thus leaving the precise identification of the sequence of galactosyltransferases action undetermined. The backbone has four N-acetyl groups on three sugar residues, a feature which may drive crucial intra- or inter-chain hydrogen bonds formation and may introduce steric hindrances. On the other hand, ketal-pyruvylation on the side chain adds a ring on both β-galactopyranose residues resulting in bulky substitution and increase of hydrophilicity due to two negative charges.

The bulky and expanded EpsA-O spatial structure in solution is consistent with the measured high intrinsic viscosity, which is comparable only to xanthan among microbial polysaccharides^50^. The high inter-chain connectivity enables gel formation at relatively low polysaccharide concentrations. The primary structure of the EpsA-O dictates its higher-order structure in solution, which in turn drives the supramolecular network formation that entraps bacteria in the gel-like biofilm. The simulations of supramolecular structure indicate that EpsA-O can span the space between neighboring bacteria in the biofilm acting as an adhesive. This study is unique as the structure of EpsA-O was studied over several orders of magnitude from atomic size to the size of the entire biofilm system. The design of EpsA-O for its adhesive function becomes evident when comparing it to levan, another extracellular polysaccharide produced by *B. subtilis*. Levan, a linear uncharged homopolysaccharide with two-orders of magnitude lower intrinsic viscosity, forms only weak gels with an elastic modulus several orders of magnitude lower than EpsA-O^51^. Although levan can be present in biofilms in much larger quantities^24^ than EpsA-O it acts predominantly as a filler^51,52^. On the other hand, EpsA-O is a biological structural polymer that enables the formation of the complex 3D architecture of the biofilm. From rheological data, and assuming that bacteria are connected longitudinally through space by EpsA-O (2000 nm × 190 nm × 190 nm), the total cohesive energy was calculated to be 2 × 10^−20^ J. To overcome this cohesive energy and irreversibly deform inter-chain connections, the bacterium has to be moved for at least 50 nm, which requires a force of 5 × 10^−13^ N. This force exceeds a single bacterium weight (Fg = 10^−14^ N) by 50-fold, indicating that EpsA-O inter-chain forces above the sol-gel transition could easily withstand the gravity force allowing the formation of a complex 3D biofilm structure. The question that remains to be solved is how EpsA-O is connected to the cells. One possibility is through TasA, the extracellular protein that is anchored to the bacteria via TapA^53^. In fact, TasA mutants have a significantly decreased capacity to form floating biofilm even in the presence of EpsA-O^24,25^. Similarly, TasA/EpsA-O double mutant, while initially attempting to form a biofilm, is unable to reinforce it due to the missing structural polymers^25^. The consequence is a biofilm not resilient to shear stress that rapidly disintegrates. Also, the rheology results of biofilms grown on agar^54^ are in line with these observations. The Gˊ drops significantly in Δ*eps* mutant compared to the wt biofilm. In addition, the results of purified EpsA-O gels or cells and EpsA-O mixtures indicate that the interaction of EpsA-O with other components can be only partially recovered in a mixture of EpsA-O and cells.

In conclusion, the structure of the branched RU of EpsA-O was established by composition and linkage analyses, and NMR spectroscopy. The main RU is composed of the backbone [→3)-β-d-Qui*p*NAc4NAc-(1→3)-β-d-Gal*p*NAc-(1→3)-α-d-Glc*p*NAc-(1]_n_ and the trisaccharide side chain β-d-Gal*p*(3,4-*S*-Pyr)-(1→6)-β-d-Gal*p*(3,4-*S*-Pyr)-(1→6)-α-d-Gal*p*-(1→ linked to C4 of the GalNAc residue. Up to 1/3 of the RU have a side chain lacking one pyruvylated Gal. This sequence is unique in the microbial polysaccharide world and contains the *N,N’*-diacetyl derivative of the prokaryote-specific sugar bacillosamine (QuipNAc4NAc), 4 N-acetyl and ∼1.8 pyruvyl groups. It is difficult to compare the structure presented with the results obtained by previous studies because only sugar composition or glycosidic linkages were determined, while NMR spectroscopy was not used. The studies also differ for the material investigated: cell lysates to check for intracellular PNAG with ELISA assay^26^ and Eps isolated from liquid culture^29^. The presented structure of EpsA-O partially agrees with the composition proposed by Chai et al. ^28^, who isolated the Eps from pellicles using mild sonication and found Glc, GalNAc, and Gal.

EpsA-O has very high intrinsic viscosity comparable only to xanthan^50^. Simulation of the EpsA-O structure in solution using the String-of-beads model demonstrated that the polysaccharide can span the space between neighboring bacteria in the biofilm. The supramolecular structure of EpsA-O provides a strong network in the intracellular space that enables gel formation and production of a 3D biofilm architecture. Using different molecular, mechanical and biophysical tools we provide a unique vantage point that spans several orders of magnitude, from atomic to system level in biofilm structure formation that will stimulate further research.

## Methods

### Bacterial strains and growth conditions

*Bacillus subtilis* subs. subtilis NCIB 3610 WT (undomesticated prototroph)^55^ and its derivative strains were used in this study. The *tasA sinR* double mutant (∆*tasA*::*spec* ∆*sinR*::*kan*; here referred as ∆*sinR*)^23^ and *tasA eps* double mutant (∆*tasA*::*spec* ∆*epsA-O*::*tet;* here referred as ∆*eps'*) were kindly provided by Roberto Kolter^56,57^, while strains TB501 (*comI amyE*::P_*hyperspank*_-*mKate* (Spec) here referred as WT) and TB525 (*comI eps*::*tet amyE*::P_*hyperspank*_-*mKate* (Spec) here referred as ∆*eps*) were kindly provided by Ákos T. Kovács^58^. Overnight cultures were grown in LB liquid medium (tryptone 10 g/L; yeast extract 5 g/L; NaCl 5 g/L) containing spectinomycin (100 μg/mL), tetracycline (10 μg/mL) or kanamycin (5 µg/mL) at 37 °C with shaking (200 rpm) for 16 h, when the cultures reached OD_650_ ~ 2.0 AU. For the selection of optimal growth medium, three different liquid growth media LB, TY^29^ and MSgg^15^ with or without supplements (galactose 0.5% (w/v); glucose 1% (w/v); MSgg with increased glycerol concentration to 12.5 g/L) were inoculated and incubated for 24 h at 37 °C at 200 rpm. The EpsA-O was isolated without the homogenization step (see Isolation of EpsA-O polysaccharide).

For EpsA-O isolation, the biofilms were grown on solid MSgg agar plates with an increased concentration of glycerol (optimal growth medium): 100 mM MOPS (3-(N-morpholino) propane sulfonic acid); 5 mM K_3_PO_4_; 50 mg/L tryptophan; 50 mg/L phenylalanine; 2 mM MgCl_2_∙6H_2_O; 0.5% (w/v) sodium glutamate; 1.25% (w/v) glycerol; 700 μM CaCl∙2H_2_O; 50 μM FeCl_3_∙6H_2_O; 50 μM MnCl_2_; 1 μM ZnCl_2_; 1.5% agar; 2 μM thiamine hydrochloride. The pH was adjusted to 7.0. All components of the medium, except thiamine hydrochloride, were autoclaved together at 110 °C. On each MSgg agar plate with a diameter of 90 mm, 250 µL of the overnight culture (Δ*sinR* or Δ*eps* strain) was inoculated in order to obtain continuous biofilm formation. The agar plates were incubated facing up at 37 °C for 24 h in a cardboard box covered with aluminum foil, following the isolation procedure (see Isolation of EpsA-O polysaccharide).

For microscopy, the overnight cultures were grown in LB liquid medium and incubated at 28 °C for 24 h. Biofilms were grown on solid MSgg agar in custom 3D printed 8-well chambered slides (Supplementary Fig. 16). Each well contained 300 µL of MSgg agar with a standard concentration of glycerol (0.5% (w/v)). The overnight cultures (WT, ∆*eps*) were diluted 1:64 by MSgg liquid medium and 4 µL inoculum was transferred to each agar well. To obtain continuous biofilm each inoculum was spread by a glass bead and incubated in climatic chamber ICH260L (Memmert, Germany) at 37 °C, 80% RH humidity for 15 h. After this time biofilm mass did not increase, indicating the biofilm was in the mature state.

For biofilms with exogenous addition of EpsA-O, the same growth conditions were used, except MSgg media or 0.2% exogenous EpsA-O was added at three stages: at inoculation, and after 4 and 7 hours of incubation. The 0.2% concentration ensured that the EpsA-O polysaccharide remained below the critical level.

### Microscopy of *Bacillus* biofilms

After 15 h of incubation the 8-well slides with grown biofilms were observed under an inverted microscope Axio Observer Z1, LSM 800 (Zeiss, Germany) operated by ZEN 2.6 software. To obtain bright-field images of the biofilm surface, the 8-well slides were observed without cover slip by an air objective LD Plan-Neofluar 20×/0.4 Korr M27. The images were acquired by AxioCam MR Rev3 (Zeiss, Germany), with the resolution of 924×924 pixels. For WT biofilms that showed pronounced surface undulation the images were acquired using extended depth of focus function of ZEN software. To obtain an insight of the biofilm structure, confocal laser scanning module (LSM 800) was used. For this purpose, the 2–6 µL of MSgp (MSgg, where glycerol is replaced by pyruvate) was added on top of the biofilms grown in 8-well slides that were carefully covered by glass cover slip, without touching the biofilm surface. The pinhole size of LSM 800 was set to 1 AU, and the sampling rate in Z-axis was set to by software software-recommended Nyquist rate. The image resolution was set to by ZEN recommended 1610 × 1610 pixels. The images were acquired by LD LCI Plan-Apochromat 40×/1.2 Imm Korr DIC M27 silicon oil objective. The acquired confocal image stacks were deconvolved by ZEN software. The 3D reconstructions were obtained by maximum intensity method in the ZEN software. The same LSM 800 settings and 8-well slide preparations were made for observation of biofilms with exogenous addition of EpsA-O. At least 4 independent biofilms were observed.

### Visualization of EpsA-O by microscopy

For EpsA-O visualization, *B. subtilis* NCBI 3610 strains Δ*eps*, Δ*sinR*, and wt were grown in MSgg liquid media at 37 °C with shaking (200 rpm) overnight. 2 µL of overnight culture was mixed with an equivalent volume of Indian ink (Royal Talens, Netherlands) on a microscope slide. A cover glass was placed on the slide glass and any excess fluid was pushed out using thumb pressure^59,60^. The negatively stained samples were observed by Plan-Apochromat 100×/1.40 Oil DIC M27 oil objective. The presence of EpsA-O was indicated by the exclusion of the Indian ink granules around the bacterial cell or bacterial aggregate. Five independent experiments were performed.

### Isolation of EpsA-O polysaccharide

After 24 hours of incubation, the biofilm was scraped from the MSgg agar plates by microscopy slide and resuspended in a 10-fold volume of saline solution. An Ultra-Turrax T8 homogenizer (set to level 4) was used to achieve an optimally re-suspended sample. Samples were then aliquoted into 1.5 mL cooled centrifuge tubes (7 mL of the sample) and sonicated for 5 seconds per mL with an amplitude of 12 µm using an ultrasonic disintegrator with a 3 mm exponential tip (MSE Scientific Instruments). The distribution of samples allowed us to treat all samples under the same conditions and with maximum efficiency. After sonication, samples were stored in ice and NaOH was added to 0.1 M final concentration. The samples were well stirred and incubated for 5 min at room temperature, then stirred again and incubated on ice for an additional 5 min, before adjusting the pH to 7.00 ± 0.02 using cooled HCl. This was followed by centrifugation for 40 min at 12000 rcf at 4 °C. To the supernatant cooled isopropanol was added (1:3 V/V) and incubated at 4 °C overnight. The precipitate was recovered by centrifugation 12,000 rcf at 4 °C for 20 min, and dried for 36 h at 55 °C to yield the raw isolate which was further purified. To obtain purified EpsA-O, the precipitates were further purified without prior drying.

### Purification of EpsA-O polysaccharide

The raw isolate of EpsA-O polysaccharide from Δ*sinR* strain was purified with dialysis membrane and by changing the pH of the solution. Based on the estimated Mw of impurities, determined by high-pressure size exclusion chromatography (HPSEC), appropriate dialysis tubing membranes were used. The purification of the raw isolate was performed in several steps. First, the raw isolate was resuspended in deionized water and isopropanol in 1:1 ratio. In this way, contamination of the samples during the purification procedures as well as contamination by microorganisms that could degrade the polysaccharide were prevented. The sample solutions were then transferred to a dialysis membrane with the cut-off of 300 kDa (Biotech, CE) and dialyzed against dH_2_O for 48 h. Then, NaOH was added to the dialysate, pH was adjusted to 12, and transferred into a fresh dialysis membrane sack with a pore size of 300 kDa. Dialysis was performed in 10 times the volume of NaOH with pH 12 for two hours. The reason for the short exposure to high pH is the instability of the dialysis membrane. The dialysis membrane was then transferred to 300 times the volume of dH_2_O and dialyzed for 4 days with constant mixing. Distilled water was replaced with fresh dH_2_O every 20 hours. After the final step of dialysis, the entire procedure was repeated once more. That is, the dialysate was again exposed to NaOH solution with pH 12 and dialyzed against NaOH (pH 12) for two hours. Afterwards, the dialysis membrane containing the sample was dialyzed against distilled water for 3 more days.

Once the desired purity of the polysaccharide was achieved, the purified isolate was frozen at −80 °C. After 2 h at −80 °C, the samples were transferred to a pre-cooled ScanVac CoolSafe freeze dryer (LaboGene, Denmark) and lyophilized for 36 h at 0.1 mbar and −95 °C.

The purity of the isolate was checked by UV-VIS spectroscopy, HPSEC and by determining the possible protein contaminants by Bradford reagent as described by Dogsa et al. ^24^. In addition, DNA gel and SDS-PAGE electrophoresis of purified EpsA-O samples were performed. For DNA gel electrophoresis, 0.8% (w/v) agarose gel in 1× TAE buffer was prepared. Samples of EpsA-O polysaccharide (0.1% and 0.2%, diluted in dH_2_O) and isolated genomic DNA from *B. subtilis* PS-216 were mixed with loading dye (6x TriTrack – Thermo Scientific) at a 3:1 (v/v) ratio and loaded onto the gel. A standard DNA ladder (GeneRuler DNA Ladder Mix – Thermo Scientific) was added to the first well. Electrophoresis was conducted for 60 minutes at 75 V. The gel was then stained with GelRed dye for 25 minutes and rinsed with dH_2_O. Gels images were captured using a G-box (SynGene, UK). For SDS-PAGE electrophoresis, precast gels (Mini-PROTEAN TGX Gels – Biorad) were used. BSA at different concentrations (0.05%, 0.10%, 0.15%, 0.20% w/v) was used as standard. Samples of EpsA-O polysaccharide were prepared at concentrations of 0.1% and 0.2%, diluted in dH_2_O. To 25 μL of each sample, 23.75 μL of Laemmli buffer and 1.25 μL of 2-mercaptoethanol were added. The mixtures were then vortexed and incubated in a thermoblock for 8 minutes at 107 °C. After incubation, 10 μL of the cooled samples were carefully pipetted into the gel wells. Two wells were loaded with the standard (PageRuler Prestained Protein Ladder, Thermo Scientific) to determine the molecular weight (Mw) of the proteins. The gel was then placed in a buffer tank, filled with 1x SDS-PAGE running buffer, and run for 30 minutes at 200 V. After electrophoresis, the gels were stained in 40 mL of staining solution (12 mL distilled water, 20 mL methanol, 4 mL acetic acid, and 4 mL of 0.1% Coomassie Brilliant Blue dye) for 30 minutes. Destaining was carried out for 30 minutes in 40 mL of destaining solution (20 mL distilled water, 16 mL methanol, and 4 mL acetic acid) and repeated three times. Finally, the gels were imaged using a G-box (SynGene, UK).

### Reducing sugars quantification, pyruvylation degree estimation and EpsA-O concentration determination

The quantification of reducing sugars was attempted by DNS assay^61^. For calibration curve, glucose solutions in concentrations from 0 to 5 mM were used. DNS reagent and standards with known concentrations were mixed in 1:1 volume ratio. The samples (raw isolates from Δ*eps* and Δ*sinR* strains, purified EpsA-O polysaccharide isolate from Δ*sinR* strain) were dissolved in dH_2_O at a concentration of 0.5% (w/v) and then prepared in the same way as standard solutions for measuring the amount of reducing sugars. The prepared samples were incubated in a thermoblock set at 112 °C for 20 min. The temperature in the test tubes was 100 °C ± 2 °C. 300 µL of each sample was transferred into 96-well microtiter plate. This was followed by absorbance measurement with Multiscan reader (Thermo Scientific, USA) at a wavelength of 575 nm.

The degree of pyruvylation was determined as described by Pinto et al. ^62^. Briefly, for the calibration curve various dilutions of sodium pyruvate (0%, 0.005%, 0.01%, 0.02%, 0.04%, 0.06%, 0.08%) were prepared. An aliquot (1 mL) of each sample was transferred to a 15 mL centrifuge tube, 1 mL of 2,4-dinitrophenylhydrazine (0.5% in 2 M HCl), and 1 mL of dH_2_O added and the mixture incubated for 10 minutes in a 37 °C water bath. After incubation, 5 mL of 0.6 M ammonium hydroxide was added. The hydrolysis of the samples was performed using 0.5% EpsA-O solution in 2 M HCl for 5 hours at 100 °C. Then, 2 mL of the hydrolyzed material was transferred to a 15 mL centrifuge tube and 9 mL of distilled water was added. After that, 1 mL of different samples was mixed with 1 mL of 2,4-dinitrophenylhydrazine (0.5% in 2 M HCl) and 1 mL of dH_2_O, and incubated the mixture for 10 minutes at 37 °C, followed by the addition of 5 mL of 0.6 M ammonium hydroxide. The absorbance was measured at 420 nm.

The EpsA-O content of dry raw isolate was determined using a modified phenol-sulfuric acid method^63^, with samples incubated for 20 min at 100 °C. For the calibration curve, the purified EpsA-O isolate was dissolved at 0.5 mg/mL, 1 mg/mL, and at 3 mg/mL in dH_2_O. For these analyses at least three replicates were made.

### High-performance size exclusion chromatography

The pullulan standards (PSS Polymer Standards Service, GMBH, Germany), raw isolates, and purified isolate were dissolved in 0.05 M NaOH to obtain a final concentration of 0.2% (w/v). All samples were heated in a thermoblock for 30 min at 55 °C in order to reduce the concentration of dissolved gases and improve the solubility of the polymers. A PSS SUPREMA GPC/SEC column was used in combination with a PSS SUPREMA pre-column (PSS Suprema Analytical, Germany). On-line detection was carried out by UV absorbance using a K-2501 detector (Knauer, Germany) at 260 nm and by Smartline RI 2300/2400 detector (Knauer, Germany). The mobile phase consisted of 0.05 M NaOH at pH 12.5. All measurements were obtained at 35 °C using a mobile phase flow of 1.00 mL/min and a sample loop of 112 µL.

### Viscoelasticity of EpsA-O polysaccharide and EpsA-O polysaccharide - cells mixtures

To determine the viscoelasticity, two dynamic moduli depending on the strain were measured with a modular oscillating rheometer Anton Paar Physica MCR 302 with a cone plate-plate (CP50-1, *d* = 50 mm) measuring system. The measurements temperature was kept at 20.0 °C. The elastic modulus (G’) measures the elastic response of the material, which measures stored energy, while the viscous modulus (G”) measures the viscous response of the material, where energy is dissipated as heat. The viscoelasticity of the polysaccharide solutions was measured at a constant frequency of 10 rad/s and by increasing the strain from 5 to 1000%. During the measurement, 17-19 logarithmically spaced measurement points were acquired.

To prepare the mixtures of EpsA-O polysaccharide and cells, biofilms of the Δ*eps* strain on MSgg agar plates were grown for 24 hours. The biofilm (100 mg) was scraped into a microcentrifuge tube and 300 μL of 2 mg/mL EpsA-O polysaccharide solution were added. For the cell sample, we mixed 100 mg of biofilm (cells) with 300 μL of dH_2_O and for the EpsA-O sample, we mixed 100 mg of dH_2_O with 300 μL of 2 mg/mL EpsA-O. The EpsA-O was added at this concentration to ensure it remained below the critical level. The samples were placed in a desiccator to concentrate the polysaccharide until only 100 μL remained in the tube. Finally, the rheological properties were examined as described above. For all viscoelastic tests at least three independent measurements were performed.

### Molecular mass determination of EpsA-O

The MM of EpsA-O was determined by universal calibration method^64^. To construct the calibration curve, the intrinsic viscosity, [η], and elution volume, V_e_, on HPSEC of pullulan standards with known MM dissolved in 0.05 M NaOH were determined. The MM of EpsA-O was determined by comparing the calibration curve to the intrinsic viscosity and elution volume of purified EpsA-O in 0.05 M NaOH.

### Modeling the spatial structure of EpsA-O

To obtain the spatial structure of EpsA-O the String-of-beads model^45–47^ was upgraded in order to simulate branched, comb-like polymers.

Briefly, in this model, the polymer molecular conformation is represented as a string of *N* monomer units, which are approximated by homogeneous impenetrable hard spheres called beads, of diameter, *d*. The branching is controlled by setting the length of the main chain and side chain segments composed of beads. A specific structural situation is defined with an individual set of four shape parameters *Θp*, *Φp*, *Θplim*, and p*, where *Θp* and *Φp* represent the bond and torsion angles between the adjacent beads, respectively; p* is the probability for the random variation of the two angles, and *Θplim* is the upper limit of the bond angle in the case of its random variation. If p* = 0 all the beads in the string have constant values of *Θp* and *Φp*, leading to a helical string, otherwise the higher the value of the parameter p*, the more beads are assigned with random values of these angles. The value of the parameter p* therefore indicates the degree of randomness (dynamics) in the molecular structure, while the value of the parameter *Θplim* reflects the stiffness of the polymer chain. Due to the random steps in the growth of the modeled molecular configuration, an individual set of four shape parameters represents an infinite set of molecular configurations that are structurally alike and mimic the case of real polymers that are not static and rigid structures. Therefore, individual structures shown in the figures can be regarded as snapshots of polymer spatial structures.

By combining the data on MM of EpsA-O, as obtained by the universal calibration method, and its primary structure, it was possible to calculate *N* and the size of the main and side chain segments. The size of the monomer unit was assumed to be the size of anhydrous glucose (i.e. *d* = 0.52 nm) and having MM = 200 Da. In the absence of detailed spatial information, the EpsA-O was modeled as a freely rotating chain (p* = 1), where the chain stiffness was controlled by setting the *Θplim* to a constant value. The choice of the value of *Θplim* is, however, not straightforward. The *Θplim* controls the chain stiffness and therefore it is strongly related to the persistence length, *lp* that our model can calculate for each simulated polymer. Therefore, if the *lp* of the polymer is known, one can then obtain the value of *Θplim* by stepwise increasing *Θplim* until the desired *lp* of the polymer is obtained.

According to the model by Yamakawa et al. ^48^, the intrinsic viscosity can be predicted by knowing the persistence length, *lp*, of the linear polymer, contour length, chain thickness, and MM. The application of this model to infer *lp* of the EpsA-O, is however not straightforward, as the model is valid for linear polymers and microbial polysaccharides are often branched. The ratio of branched to linear dilute solution properties of polymers is often expressed in contraction factors, $g_{Rg^{2}}$, for the ratio of radii of gyration and *g*_*[η]*_, for the ratio of intrinsic viscosities of branched and respective linear versions of the same polymer. For the random coil polymer that is branched as a symmetrical comb, the analytical solution for $g_{Rg^{2}}$ was established by Casassa & Berry^65^. Defining the number of main chain segments to be proportional to the difference between total chain mass and side chain mass, and assuming there are many polymer branches, the expression for $g_{Rg^{2}}$ ^65^ can be simplified to:

1 $${{g}}_{{{Rg}}^{2}}=1-{\mathrm{DB}}$$

However, for our modeling *g*_*[η]*_ was needed which is related to $g_{Rg^{2}}$^66^:

2 $$g_{[\eta]}=(g_{{{Rg}}^{2}})^{\epsilon}$$

Although it is still unclear what is the exact value of ϵ in the case of comb-like polymers, there is growing evidence that *ϵ* ≈ 1^67–69^, which was assumed to be the case in EpsA-O.

The DB resulted from the EpsA-O primary structure and *g*_*[η]*_ was then calculated. Therefore, it was possible to determine the theoretical intrinsic viscosity of the linear variant of the otherwise branched native EpsA-O for which the intrinsic viscosity was measured. By using the Yamakawa model equation, the value of *lp* of EpsA-O linear variant was retrieved, and subsequently also the value of *Θplim* of simulated EpsA-O.

To determine the volume fraction of extracellular space in the WT *B. subtilis* biofilms the CLSM images of biofilms were analyzed by Fiji-ImageJ^70,71^. As the lateral resolution (i.e. x-y plane) is larger than the axial resolution in CLSM, parts of the biofilm were examined, where the fiber-like bacterial structures were oriented perpendicular to the x-y image plane in CLSM. The identified regions were enveloped by the Freehand selection tool, followed by area calculation. The number of *B. subtilis* cells that appeared as circles of 750 nm in diameter^49^ inside the region was manually counted. The volume fraction of extracellular space was then obtained as a ratio of the area of all cells divided by the total area of the enveloped region. To obtain an average and SD, six regions were analyzed.

The 3D structures and their x-z projections were plotted in OriginPro (OriginLab, Northampton, USA). For pore diameter distributions, the images were exported to Fiji-ImageJ, where the pore areas were analyzed. The diameters were calculated as the square roots of the pore areas and the distributions were obtained in OriginPro. The pore distribution shown is an average of the distribution of five projections of five independent simulations.

### Cohesive energy and forces calculations

The estimate of cohesive energy density, *E*_*c*,_ of the EpsA-O in the biofilm was calculated^72^:3where *G’* is the value of elastic modulus of EpsA-O 1% (w/v) in Msgg medium in the linear viscoelastic range, *γ*_*c*_ is the critical strain, where, beyond this point, the structure is irreversibly broken down. Based on microscopy data, the intercellular volume, occupied by EpsA-O, of two neighboring bacillus cells in a single layer of biofilm extending along a longer axis of bacillus was calculated to be 2000 nm × 190 nm × 190 nm. The cohesive energy of EpsA-O in this volume is 2 × 10^−20^ J (at *γ*_*c*_ = 25%, Fig. 4e). The distance needed to irreversibly move two cells apart is about 50 nm (25% from 190 nm). The force required to perform this work, which is equal to the cohesive energy, is therefore 5 × 10^−13 ^N.

### Composition and linkage analysis of EpsA-O

Before chemical derivatizations, EpsA-O was dissolved in water, sonicated in an ice bath with 20 bursts of 1 min each, separated by 1 min intervals using a Branson sonifier equipped with a microtip at 2.8 A, and lyophilized. Composition analysis was carried out by chemical derivatization of the sonicated EpsA-O both as alditol acetates and trimethyl glycosides. Alditol acetates were obtained as described^73^ after hydrolysis with 2 M trifluoroacetic acid (TFA) for 1 h at 125 °C. Trimethylsilyl methylglycosides were obtained by derivatization with Sylon™ HTP (Merck) after methanolysis of the EpsA-O with 3 M HCl in methanol at 85 °C for 16 h^74^. Linkage analysis, through derivatization to partially methylated alditol acetates (PMAA), was performed following the protocol developed by Harris^75^; for the reduction step, sodium borodeuteride (NaBD_4_) was used. The absolute configuration (except for QuiNAc4NAc) was established by GLC analysis of the trimethylsilylated (+)-2-butyl glycosides derivatives^76,77^. The derivatized samples were analyzed by GLC using an Agilent Technologies 6850 gas chromatograph equipped with a flame ionization detector, an HP-1 capillary column (Agilent Technologies, 30 m), and using He as the carrier gas. The following temperature programs were used: for alditol acetates 3 min at 150 °C, 150–270 °C at 3 °C/min, 2 min at 270 °C, for trimethylsilyl methylglycosides 150–280 °C at 3 °C/min, for trimethylsilyl (TMS) (+)-2-butyl glycosides 1 min at 50 °C, 50–130 °C at 45 °C/min, 1 min at 130 °C, 130–200 °C at 1 °C/min, 10 min at 200 °C, and for PMAA 1 min at 125 °C, 125–240 °C at 4 °C/min, 2 min at 240 °C. GLC-MS analyses were carried out on an Agilent Technologies 7890 A gas chromatograph coupled to an Agilent Technologies 5975 C VL MSD using the same column and the temperature programs of the GLC analysis. Values of the integrated area of the PMAA were corrected by the effective carbon response factors^78^.

### Smith degradation of EpsA-O

Smith degradation was performed as previously described^32^. Twelve mg of sonicated EpsA-O were dissolved in 9.75 mL H_2_O; 60 μL of 0.76 M NaIO_4_ and 190 μL of 2.1 M MgCl_2_ were added and the solution was incubated for 7 days at 10 °C in the dark with stirring. Glycerol was added to stop the reaction and NaBH_4_ was added to pH 10 for reduction of the aldehyde groups (16 h at 25 °C). The reaction was stopped with a dropwise addition of 50% acetic acid, the solution was dialyzed, lyophilized, and hydrolyzed with 5 mL 0.1 M TFA at 24 °C for 7 days. The acid was removed by evaporation under reduced pressure, isopropanol was added and the sample was taken to dryness; the latter procedure was repeated three times. The sample, named **EpsA-O SD**, was dissolved in 1 mL of H_2_O, taken to pH = 7.3, and recovered by lyophilization. **EpsA-O SD** was dissolved in 1.9 mL of 0.05 M NaNO_3_ and separated by size exclusion chromatography on a Bio-Gel P2 column (1.6 cm i.d. × 90 cm) at a flow rate of 6.8 mL/h. Fractions were collected at 12 min intervals. Elution was monitored using a refractive index detector (Knauer, RI detector K-2301, Lab-Service Analitica) which was interfaced with a computer via PicoLog software. Three main peaks were obtained (**SD1,** **SD2,** **SD3**) and they were desalted by medium-pressure chromatography using a Bioline chromatography system equipped with a Superdex G30 glass column (1 cm i. d. × 82 cm) connected to a Smartline 1050 pump (Knauer). Elution with water at 1 mL/min flow rate was monitored with a refractive index detector (Smartline 2300, Knauer, Lab-Service Analitica) which was interfaced with a computer via Clarity software. Fractions were collected at 30 s intervals and those belonging to the same peak were pooled, recovered by lyophilization, and subjected to 1D and 2D NMR spectroscopy investigation.

### Solvolysis of EpsA-O

Solvolysis was performed by adding TFA (1.2 mL) to 16.4 mg of EpsA-O previously sonicated as reported above. The solution was stirred at 40 °C for 1h^79^. The sample was dried under a nitrogen flux and washed three times with anhydrous methanol to remove TFA. The newly formed aldehydic functions were reduced with aqueous sodium borohydride for 16 h at RT. The reduction was then stopped with acetic acid and the sample was taken to dryness by evaporation under reduced pressure. The procedure was repeated three times after adding 10% acetic acid in methanol and three times with methanol. The dried sample was dissolved in 0.15 M NaCl and subjected to middle-pressure size-exclusion chromatography on the Bioline system previously described, using a Sephacryl S-300 HR column (1 cm id x 45 cm). Elution was carried out with 0.15 M NaCl at 1 mL/min flow rate. The selection of fractions to be pooled together was obtained by applying a fitting procedure using a Gaussian function and hypothesizing two main peaks, the degraded polysaccharide, and salt. According to the fitting curves obtained, (Supplementary Fig. 9a), test tubes were pooled in three fractions, named **SL1,** **SL2**, and **SL3**, the latter being mainly salt. Fractions **SL1** and **SL2** were then desalted using a Superdex G30 prep grade column (1 cm i.d. × 82 cm) with water as eluent at 1.5 mL/min flow rate, recovered by lyophilization, and dissolved in 10 mM, pH = 7 sodium phosphate buffer, blown with N_2_ to prevent degradation of QuiNAc4NAc. ^1^H NMR analysis showed that fraction **SL1** was less heterogeneous than **SL2** and thus it was chosen for thorough NMR investigation (Supplementary Fig. 9b).

### NMR spectroscopy

Samples subjected to NMR were exchanged twice with 99.9% D_2_O by lyophilization, dissolved in 0.6 mL of 99.96% D_2_O, and introduced into a 5 mm NMR tube for data acquisition. Spectra of all samples except **SL1** were recorded on a 500 MHz VARIAN spectrometer operating at 343 K after setting the proper pw90° pulse. 2D experiments were performed using standard VARIAN pulse sequences and pulsed field gradients for coherence selection when appropriate. HSQC spectra were recorded using 140 Hz (for directly attached ^1^H–^13^C correlations). HMBC experiments were recorded using a coupling constant of 8 Hz (for long-range ^1^H–^13^C correlations) and a relaxation time 1.2 s. TOCSY spectra were acquired using 100 ms spin-lock time and 1.0 s relaxation time. NOESY experiments were recorded with 200 ms mixing time and 1.0 s relaxation time. Chemical shifts are expressed in ppm using acetone as an internal reference (2.225 ppm for ^1^H and 31.07 ppm for ^13^C). NMR spectra were processed using MestreNova software. **SL1** spectra (1D ^1^H and 2D, COSY, TOCSY, and HSQC) were obtained using a Bruker Avance III 600 MHz NMR spectrometer equipped with a BBO Prodigy cryoprobe and processed using standard Bruker software (Topspin 3.2). The probe temperature was set at 343 K. The 2D TOCSY experiment was recorded using a mixing time of 160 ms and the 1D variants using a mixing time of 200 ms. The 1D NOESY experiments were performed using a mixing time of 300 ms. The HSQC (with multiplicity editing) experiment was optimized for J = 145 Hz (for directly attached ^1^H-^13^C correlations). 2D experiments were recorded using non-uniform sampling: 40% for homonuclear and 20% for heteronuclear experiments.

### Prediction of proteins function

Prediction of the function of enzymes encoded by the epsA-O cluster was performed using protein analysis tools available on the NCBI website. Sequence similarity was assessed using the BLASTP suite^80^ and conserved domains were identified using the CD Search Service^81^. Multiple sequence alignments were performed using the Constraint-based Multiple Alignment Tool (COBALT)^82^.

### Data presentation and statistical analysis

All the experiments were done in 3–6 independent biological replicates. CLSM micrographs and data of the most representative series are shown. The statistical analysis and data presentation were performed in OriginPro (OriginLab, USA) program. Experimental errors indicated in text or represented as error bars are given as standard deviations. For testing statistical significance, a two-tailed unpaired t-test was used. Samples showing *p*-value < 0.05 were considered statistically significantly different.

### Reporting summary

Further information on research design is available in the Nature Research Reporting Summary linked to this article.

## Supplementary information

Supplementary information

Reporting summary

## Data Availability

The authors declare that the data supporting the findings of this study are available within the paper and its Supplementary Material. Raw microscopic image data CLSM sets are available at FigShare database at 10.6084/m9.figshare.26878876. Additional data are available from the corresponding author upon a reasonable request.

## Code availability

The String-of-beads model is available from the corresponding authors upon a reasonable request.
