# Supplementary material for: Bacillus subtilis EpsA-O: A novel exopolysaccharide structure acting as an efficient adhesive in biofilms
Source: NPJ Biofilms Microbiomes. 2024 Oct 2;10:98. doi: 10.1038/s41522-024-00555-z (PMC11447030; doi:10.1038/s41522-024-00555-z)
Supplement: Supplementary file 1 — Supplementary information [file 41522_2024_555_MOESM1_ESM.pdf]

SUPPLEMENTARY INFORMATION

Bacillus subtilis EpsA-O: a novel exopolysaccharide structure acting as an efficient adhesive in biofilms

Iztok Dogsa[1], Barbara Bellich[2,#], Mojca Blaznik[1], Cristina Lagatolla[3], Neil Ravenscroft[4], Roberto Rizzo[3], David Stopar[1], Paola Cescutti[3,*]

[1] University of Ljubljana, Biotechnical Faculty, Department of Microbiology, Večna pot 111, Ljubljana, Slovenia

[2] Department of Advanced Translational Diagnostics, Institute for Maternal and Child Health, IRCCS "Burlo Garofolo", Via dell'Istria 65, 34137 Trieste, Italy

[3] University of Trieste, Department of Life Sciences, Via L. Giorgieri 1, Trieste, Italy

[4] University of Cape Town, Department of Chemistry, Rondebosch, 7701, South Africa

* Corresponding author: Department of Life Sciences, University of Trieste, via L. Giorgieri 1, Bdg C11, 34127 Trieste, Italy. E-mail address: pcescutti@units.it (P. Cescutti)

# When the experiments were performed Barbara Bellich was working at Department of Life Sciences, University of Trieste, Via Licio Giorgieri 1, Bdg. C11, 34127 Trieste, Italy.

Content of Supplementary Information:

Supplementary Figure 1 | The amount of EpsA-O raw isolate in different growth media.

Supplementary Figure 2 | HPLC-SEC, DNS analysis and purity test of isolated EpsA-O.

Supplementary Figure 3 | Amplitude sweep test of EpsA-O samples.

Supplementary Figure 4 | Analysis of the Smith degradation products of EpsA-O.

Supplementary Figure 5 | 1D NMR spectra of SD1 recorded at 70 °C and 500 MHz.

Supplementary Figure 6 | NOESY plot of sample SD1 recorded at 70 °C.

Supplementary Figure 7 | TOCSY plots of SD2 sample recorded at 70 °C.

Supplementary Figure 8 | Ring region of the SD2 HSQC spectrum recorded at 70 °C.

Supplementary Figure 9 | Analysis of EpsA-O sample after solvolysis.

Supplementary Figure 10 | Evidence that EpsA-O contains a minor glucan impurity.

Supplementary Figure 11 | 1D NOESY spectrum obtained with selective excitation of H1 of the 6-linked α-Gal at 4.92 ppm present in the SL1 fraction.

Supplementary Figure 12 | Repeating unit structure of EpsA-O.

Supplementary Data 1: Functions assignments of the genes *epsE*, *epsH epsJ* and *epsF* in the *epsA-epsO* gene cluster of *B. subtilis.*

Supplementary Table 1. Similarity level between Eps enzymes and proteins with known function obtained by BLAST alignment.

Supplementary Figure 13 | COBALT Multiple alignment of EpsF of *B. subtilis* and its homologous wbcN of *Yersinia enterocolitica* with some representatives of the GT4-CapH-like Family Protein.

Supplementary Figure 14 | Graphic Summary of BLAST alignments of EpsH with EpsJ and EpsE.

Supplementary Figure 15 | COBALT Multiple alignment of EpsI and EpsO pyruvyltransferases of *B. subtilis* with the enzymes WclU of *Klebsiella pneumoniae* and Pvg1 of *Saccharomyces pombe.*

Supplementary Figure 16 | The workflow for preparing slides for observing biofilms under a microscope.

Supplementary References

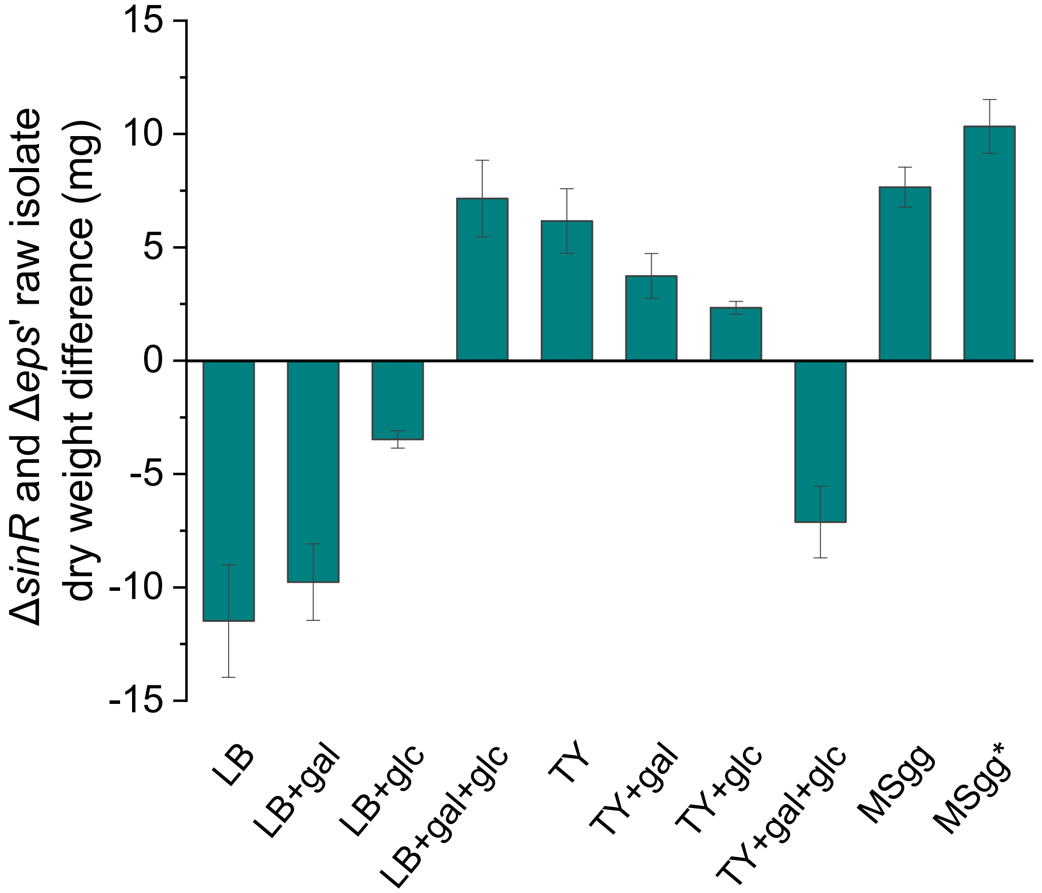

Supplementary Figure 1 │The amount of EpsA-O raw isolate in different growth media. The mass of EpsA-O raw isolate was calculated as the difference of raw isolate obtained from 12 mL of spent liquid growth-media of *B. subtilis* Δ*sinR* (EpsA-O hyper-producer) and *B. subtilis* Δ*eps'* (deficient in EpsA-O production-negative control). Three different growth media (LB, TY, MSgg) with supplements (gal = galactose 0.5 % (w/v); glc = glucose 1 % (w/v); MSgg* = MSgg with increased concentration of glycerol, from 5 g/L to 12.5 g/L) were inoculated and incubated for 16 h at 37 °C at 200 rpm, following isolation procedure (see Materials and Methods, Isolation of EpsA-O polysaccharide).

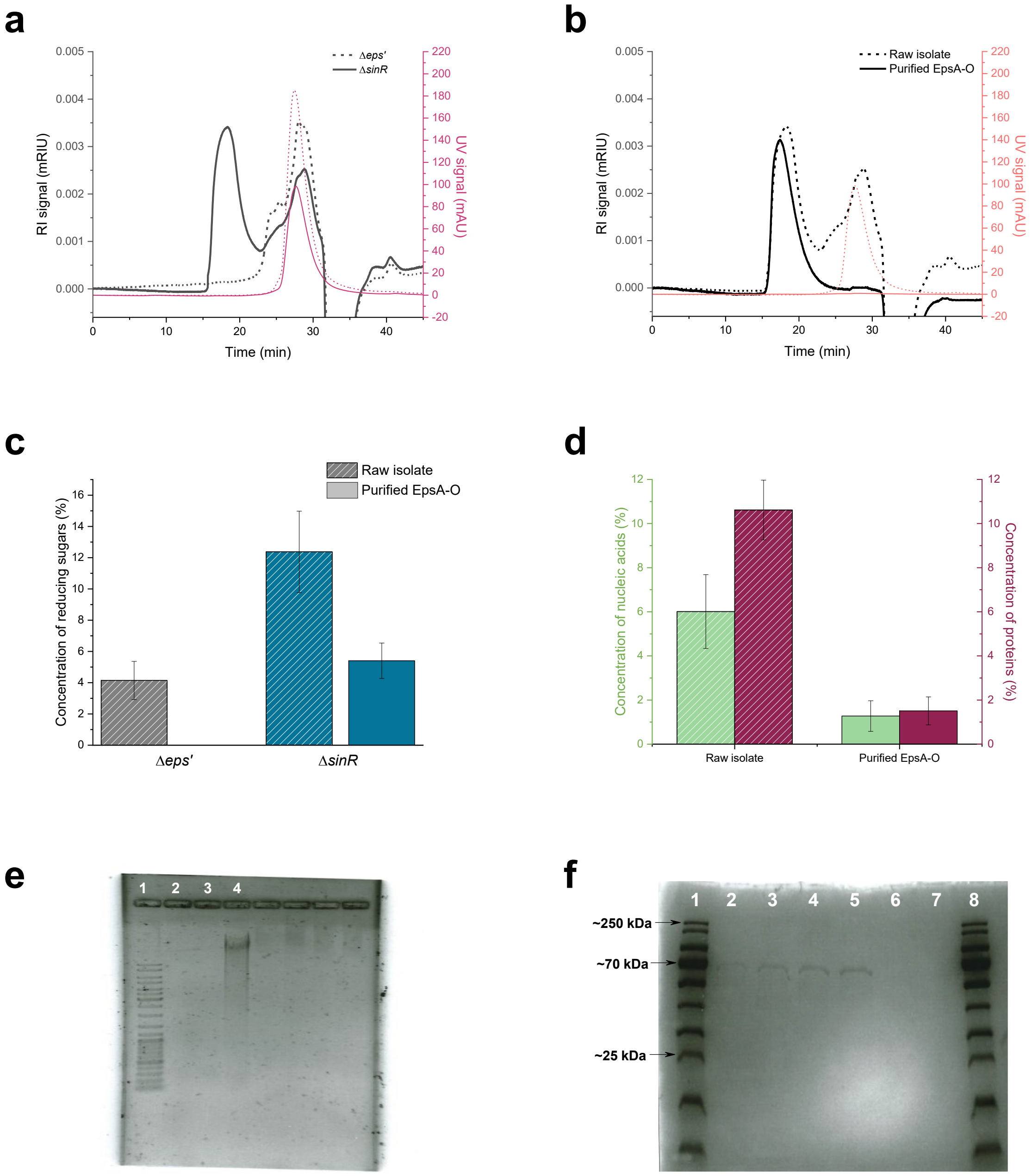

Supplementary Figure 2 | HPLC-SEC, DNS analysis and purity test of isolated EpsA-O. **a**, HPLC-SEC chromatogram recorded on RI and UV detectors of raw isolated material from Δ*sinR,* (EpsA-O hyperproducer) and Δ*eps'* (deficient in EpsA-O and TasA production). Black curves: RI detector signal (mRIU); red curves: UV detector signal (mAU). The dashed lines correspond to the *B. subtilis* Δ*eps'* strain, and the solid curves correspond to the *B. subtilis* Δ*sinR*. **b**, Δ*sinR* was further purified to obtain purified EpsA-O. Black curves: RI detector signal (mRIU); orange curves: UV detector signal (mAU). The dashed lines correspond to raw isolate, and the solid curves correspond to the purified EpsA-O. **c**, DNS analysis. Patterned bar represents raw isolates, while unpatterned bar represents purified EpsA-O sample. **d**, Concentration of nucleic acid and proteins contaminants of EpsA-O isolates in % (w/v) as determined by UV-VIS and Bradford assay, respectively. **e**, DNA gel electrophoresis of EpsA-O sample (1, DNA ladder mix; 2, EpsA-O 0.1 %; 3, EpsA-O 0.2 %; 4, *B. subtilis* PS-216 genomic DNA). **f**, SDS-page gel electrophoresis of EpsA-O sample (1, Protein ladder mix; 2, BSA 0.05 %; 3, BSA 0.10 %; 4, BSA 0.15 %; 5, BSA 0.20 %; 6, EpsA-O 0.1 %; 7, EpsA-O 0.2 %; 8, Protein ladder mix).

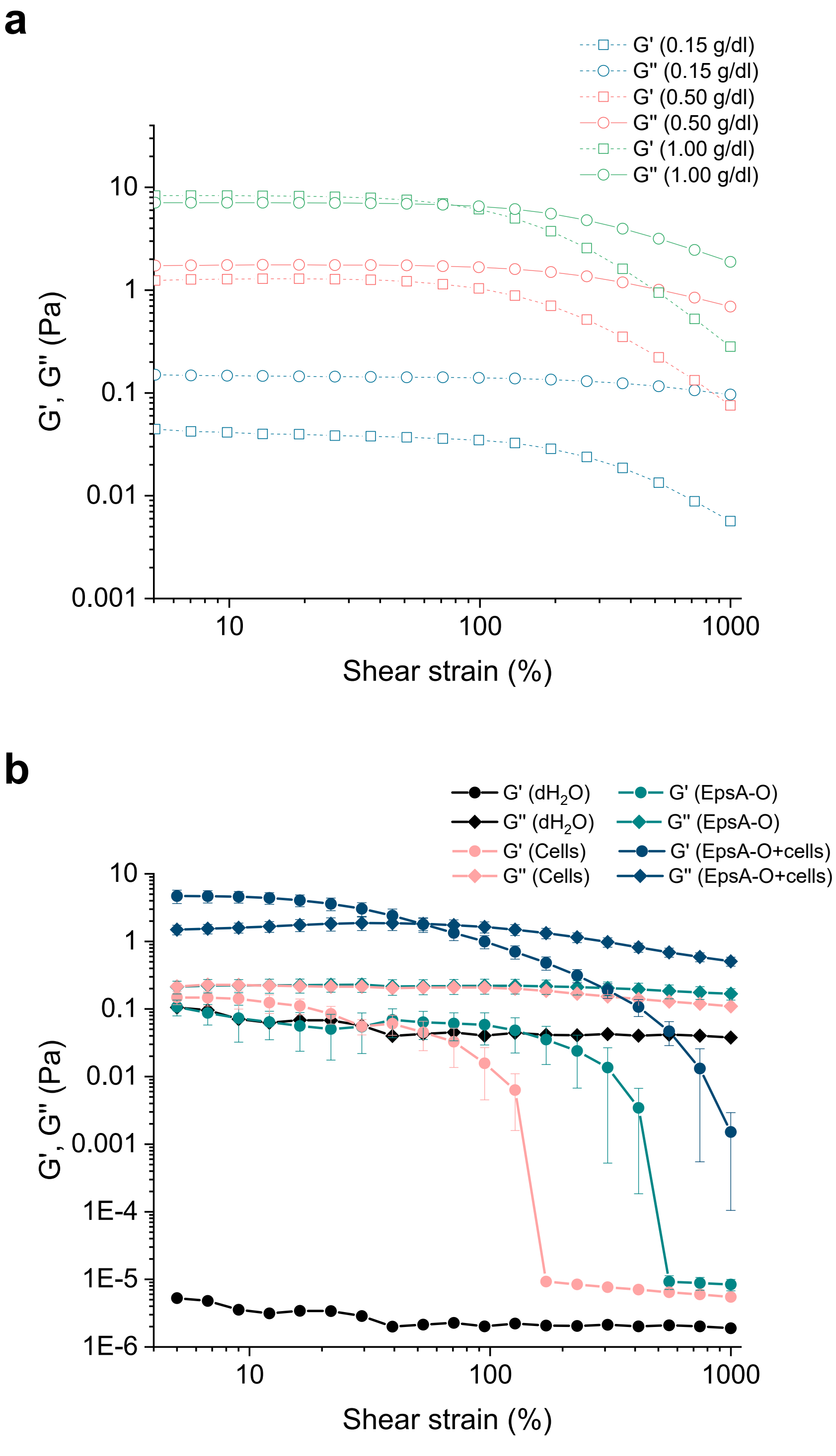

Supplementary Figure 3 | Amplitude sweep test of EpsA-O samples. a, Amplitude sweep test of EpsA-O dissolved in dH2O at different EpsA-O concentrations. Squares = storage modulus (G′); circles = loss modulus (G″). The color coding for the symbols and lines indicates different concentrations: green 1 g/dL, orange 0.5 g/dL, and blue 0.15 g/dL. **b**, The formation of a gel structure in *B. subtilis* Δ*eps* cells upon the addition of exogenous EpsA-O. An amplitude sweep test was conducted on Δ*eps* cells, Δ*eps* cells supplemented with exogenous EpsA-O, and a purified sample of EpsA-O. The final concentration of exogenous EpsA-O was 0.6 %. Circles = storage modulus (G′); diamonds = loss modulus (G″). The color coding for the symbols and lines indicates different samples: black for dH2O, orange for cells only, green for 0.6 % EpsA-O polysaccharide solution, and blue for the mixture of bacterial cells and EpsA-O in a final concentration of 0.6 %.

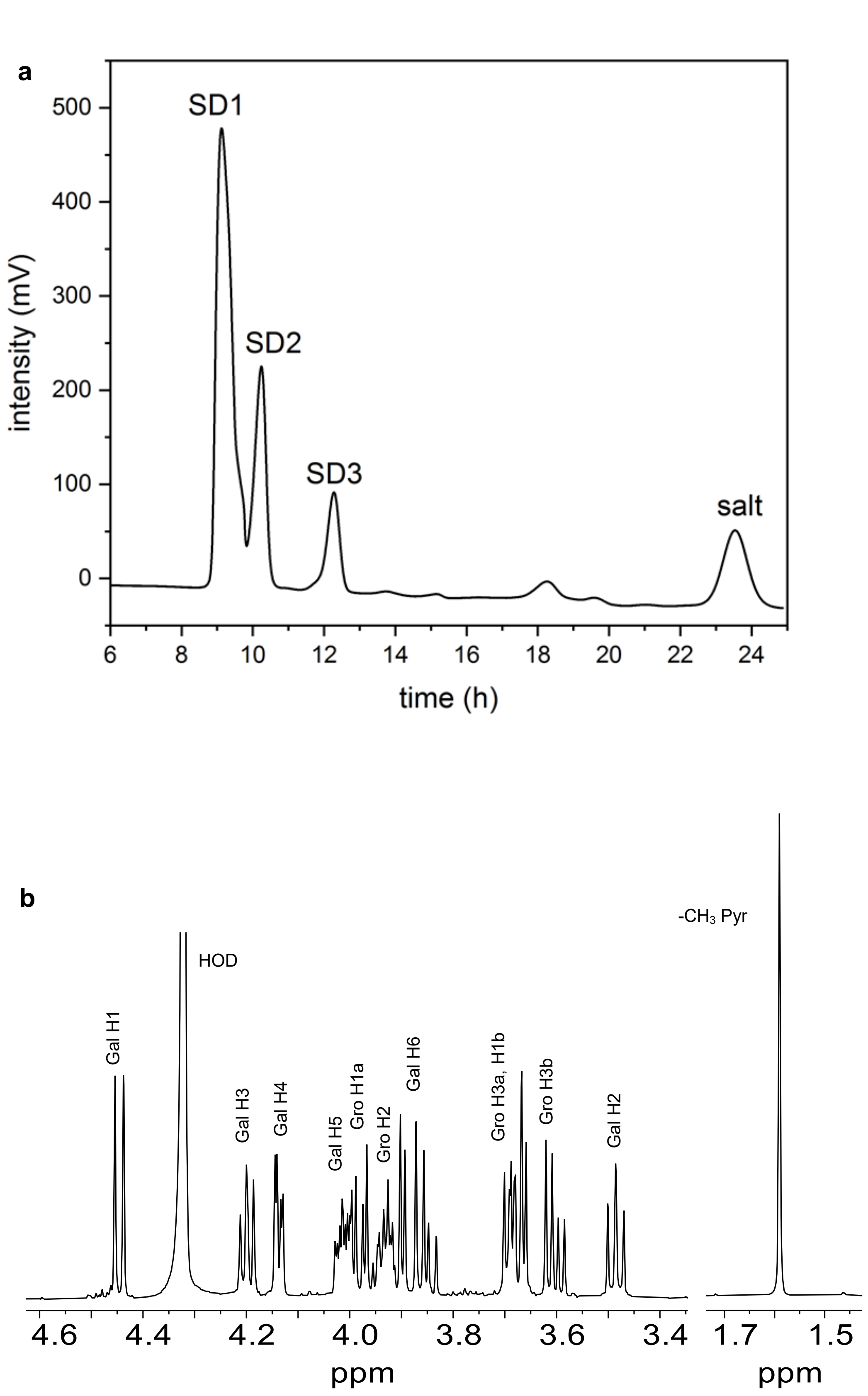

Supplementary Figure 4│Analysis of the Smith degradation products of EpsA-O. **a**, Size-exclusion chromatogram of the Smith degradation products obtained using a Bio Gel P2 column: peaks are labelled **SD1** (eluted at V_0), **SD2** and **SD3**. Integration of the **SD2** and **SD3** peak areas gave a 2:1 ratio. **b**, ^{1}H NMR spectrum of fraction **SD3** showed that it is constituted by β-D-Gal*p*(3,4-*S*-Pyr)-(1→Gro Assignments are indicated. (Gal = galactose; Pyr = pyruvyl substituent; Gro = glycerol)

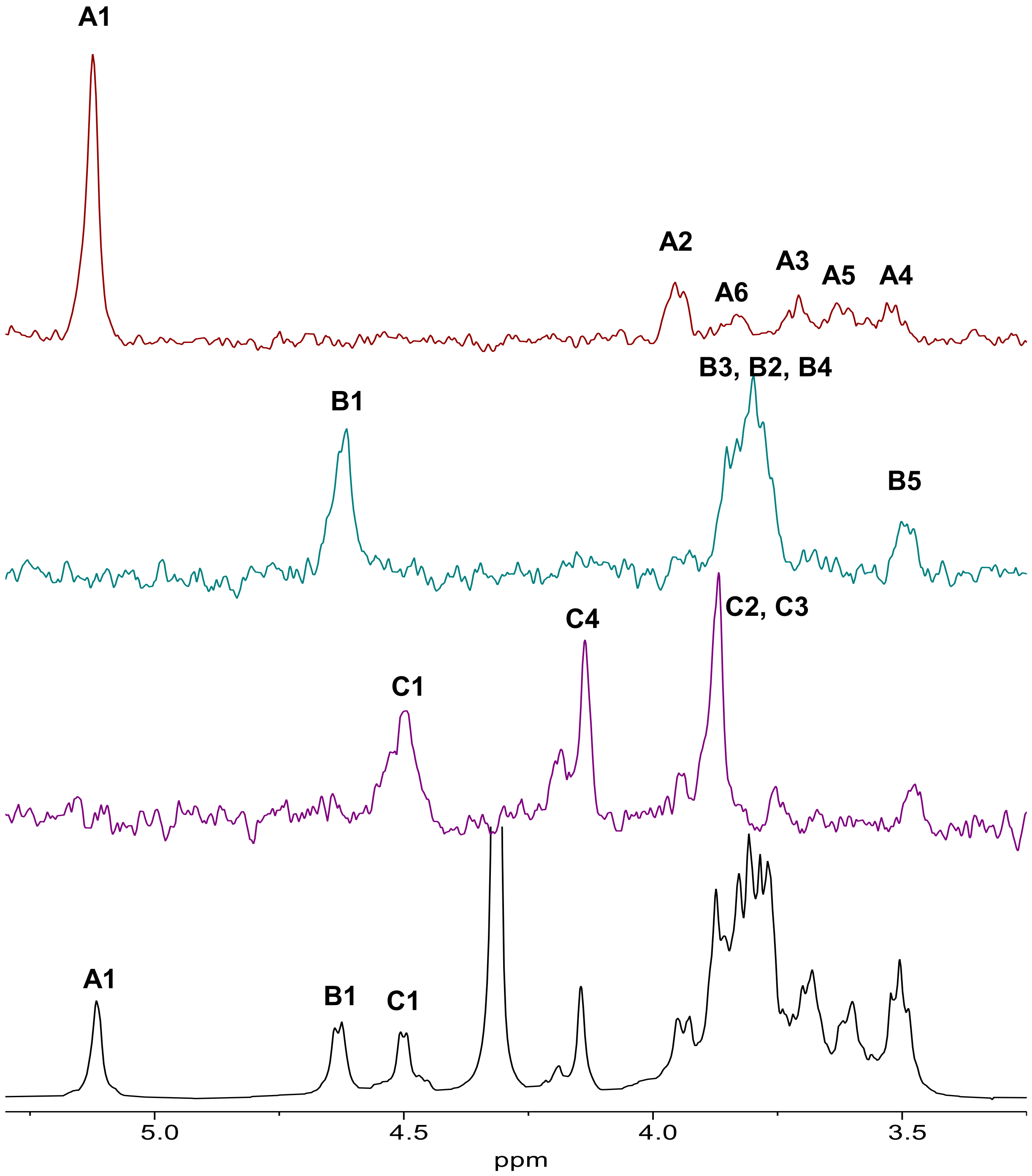

Supplementary Figure 5 | 1D NMR spectra of SD1 recorded at 70 °C and 500 MHz. 1D TOCSY (three top) and ^{1}H NMR (bottom) spectra of sample **SD1**. Peaks are labelled according to Table 1 (**A**1 = H1 of residue **A**). For **B**1 transfer of magnetization occurred up to **B**6 (not shown).

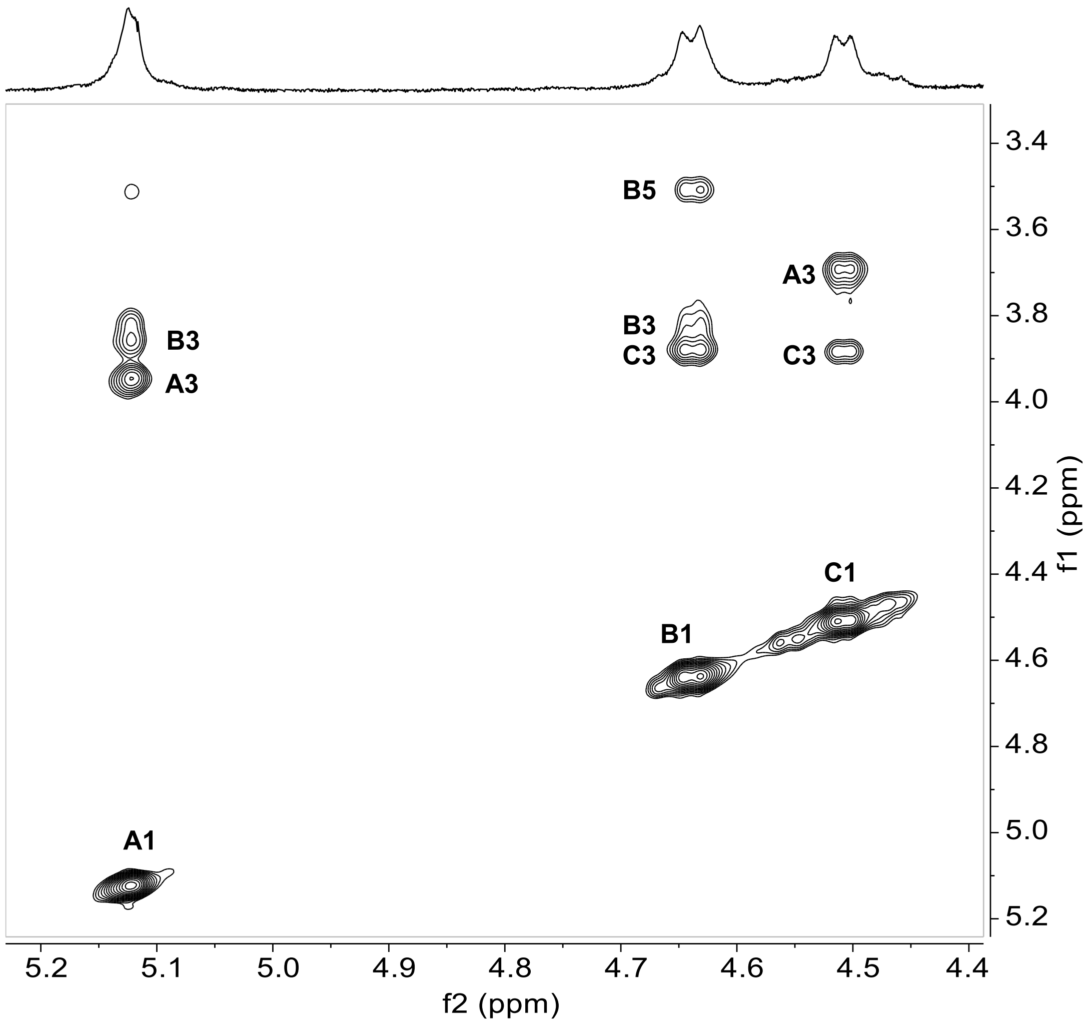

Supplementary Figure 6 │ NOESY plot of sample SD1 recorded at 70 °C. Relevant inter-residues cross peaks are labelled (**A1** = H1 of residue **A**).

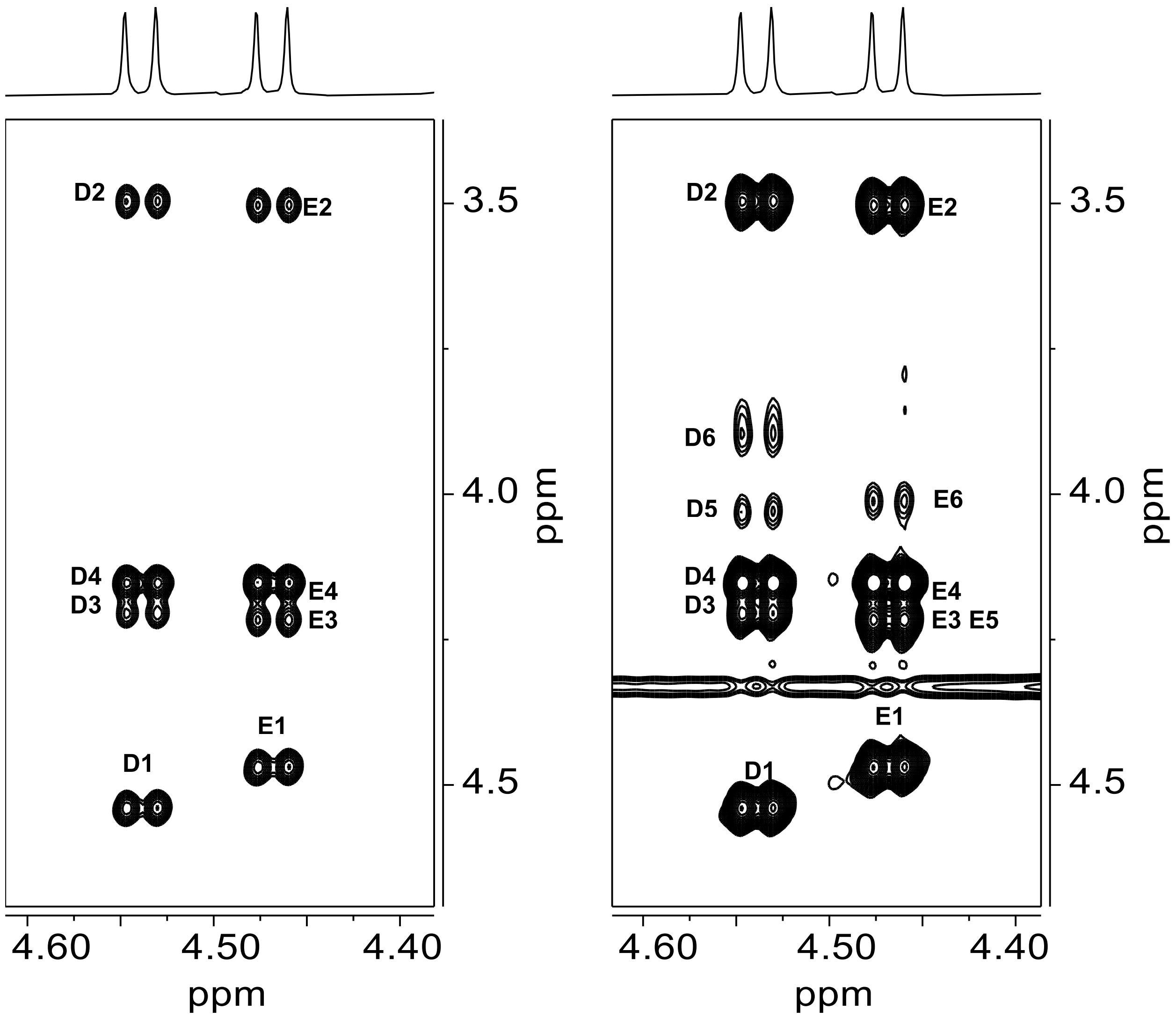

Supplementary Figure 7│TOCSY plots of SD2 sample recorded at 70 °C. Cross peaks are labelled as in Table 2 (**D**1 = H1 of residue **D**). Left, low intensity plot; right, high intensity plot showing also H5 and H6 of residues **D** and **E**.

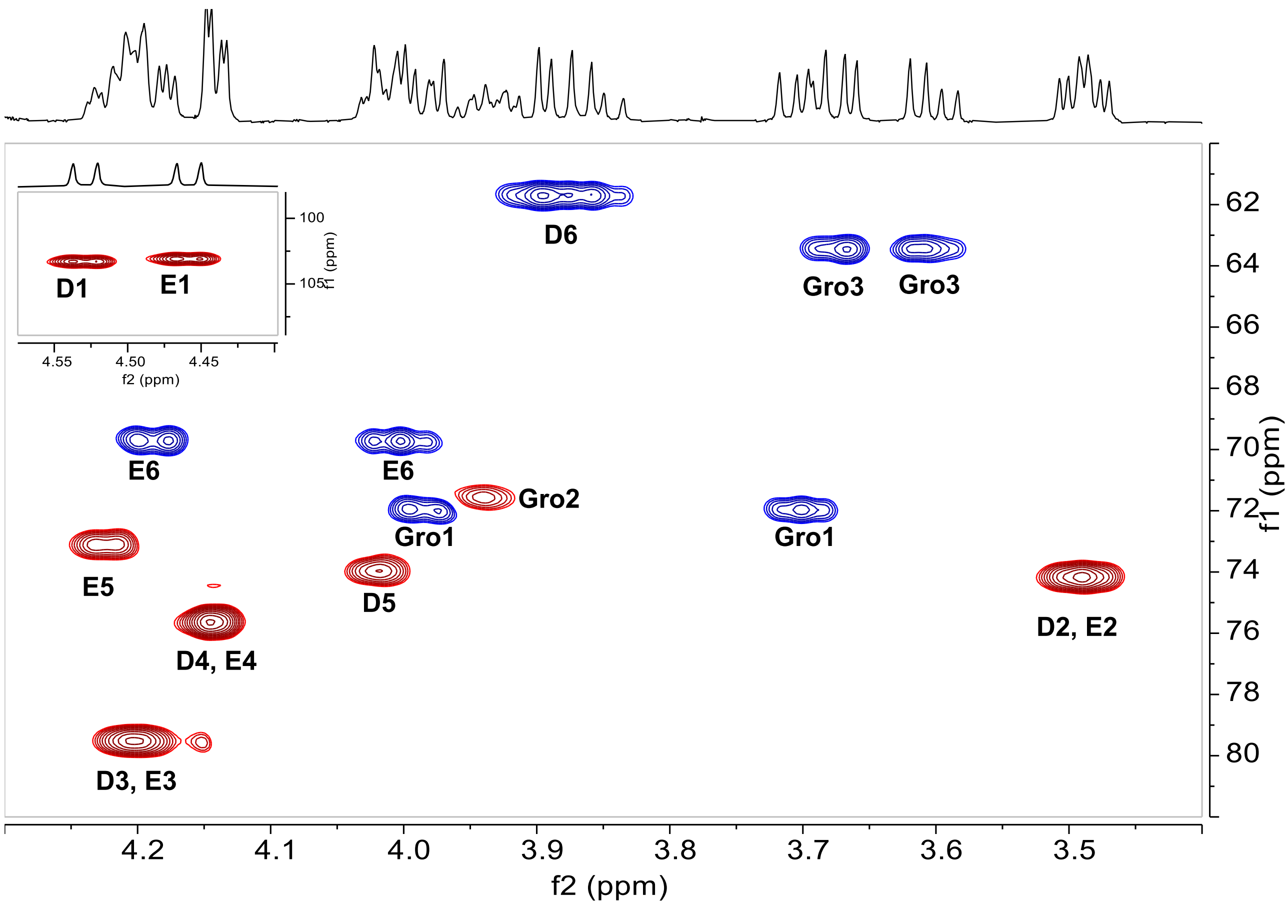

Supplementary Figure 8│Ring region of the SD2 HSQC spectrum recorded at 70 °C. The anomeric region is reported in the inset. Cross peaks are labelled as in Table 2 (**D**1 = H1/C1 of residue **D**).

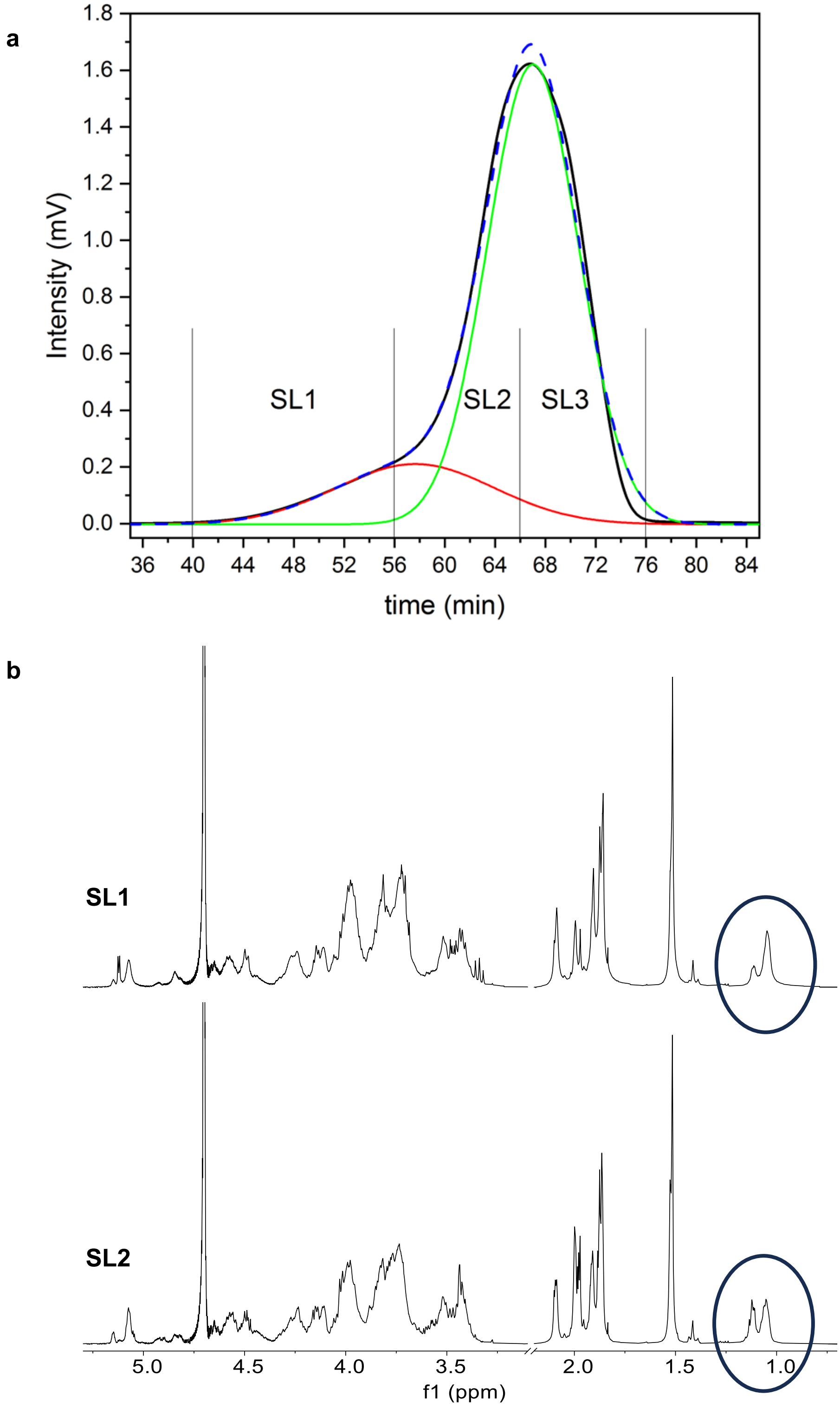

Supplementary Figure 9 | Analysis of EpsA-O sample after solvolysis. a, Chromatographic profile of EpsA-O after solvolysis in TFA (black) obtained on a S-300 HR column (1 cm i. d. X 45 cm) using a Bioline chromatographic system (see Methods). Elution was performed with 0.15 M NaCl at 1 mL/min. Green and red curves were obtained by applying a fitting procedure using a Gaussian function and hypothesizing two main peaks containing the degraded polysaccharide and salt; the dashed blue curve represents the cumulative fit; bars define which fractions were pooled together according to the fitting. **b**, ^{1}H NMR spectra of samples **SL1** and **SL2** recorded at 500 MHz and 25 °C. Circles indicate the methyl group (H6) resonance of QuiNAc4NAc which appears as two distinct peaks of equal intensity in **SL2** indicating a higher degree of structural heterogeneity of this sample.

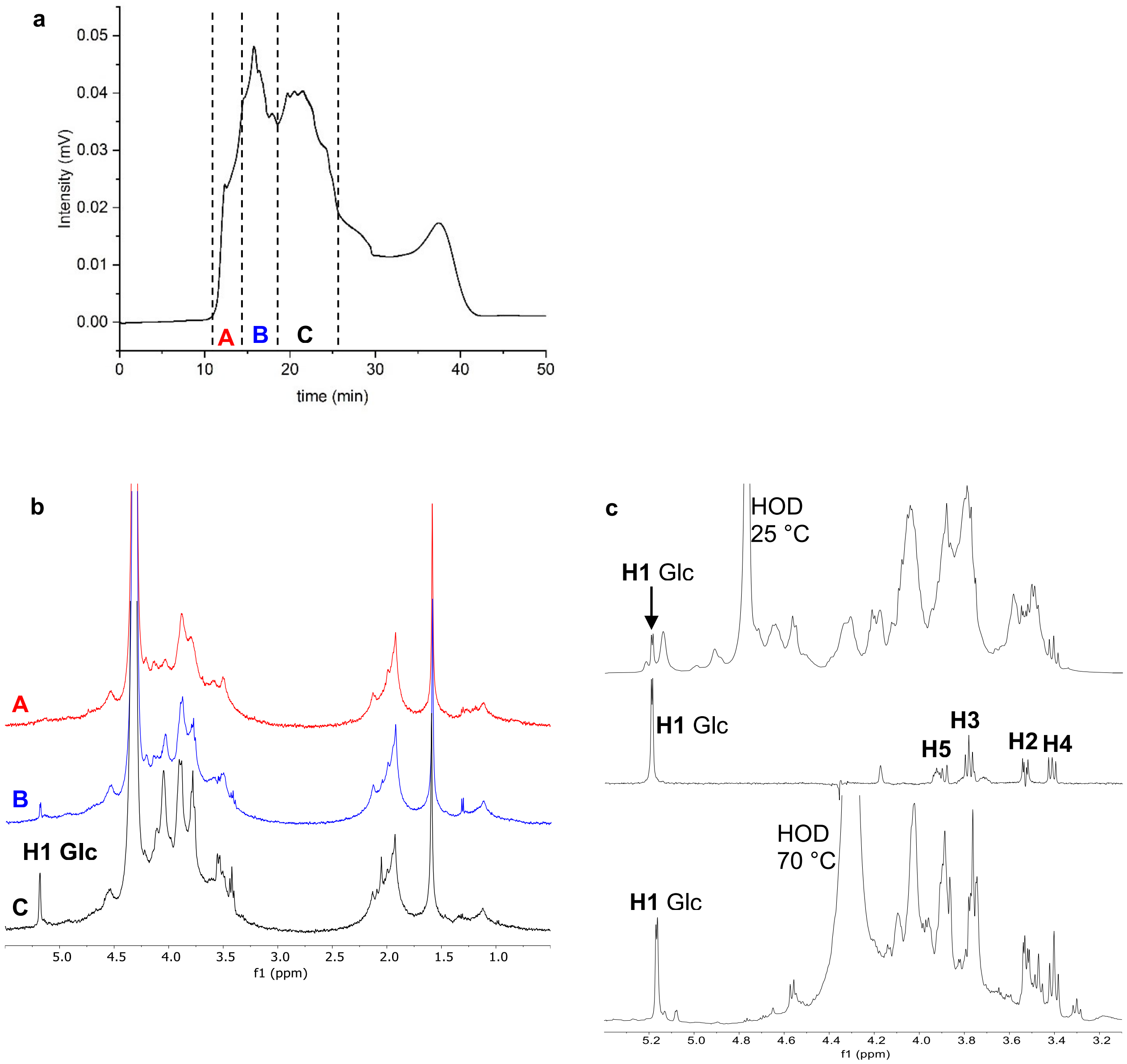

Supplementary Figure 10 | Evidence that EpsA-O contains a minor glucan impurity. a, Size exclusion chromatography profile of the native EpsA-O separated on a Sephacryl S-300 HR column (1 cm i. d. X 45 cm) using a Bioline chromatographic system (see Methods). Elution was performed with 0.15 M NaCl at 1 mL/min and fractions were collected at 30 s interval. Fractions between bars were pooled (A, B, C), desalted, exchanged with D_2O and subjected to ^1H NMR spectroscopy. **b**, ^1H NMR spectra of the fractions A, B and C showing that the intensity of signal H1 of glucose (H1 Glc) increases as the fractions MM decreases, thus proving that it is not part of the EpsA-O polymer. **c**, ^1H NMR spectra of **SL1** sample (top), the polysaccharide produced by a Δ*eps'* mutant (bottom) where the resonances related to the EpsA-O are absent, while the signal of H1 of glucose is very intense, and the 1D TOCSY spectrum (center) obtained upon excitation of H1 of glucose at 5.19 ppm present in the **SL1** fraction.

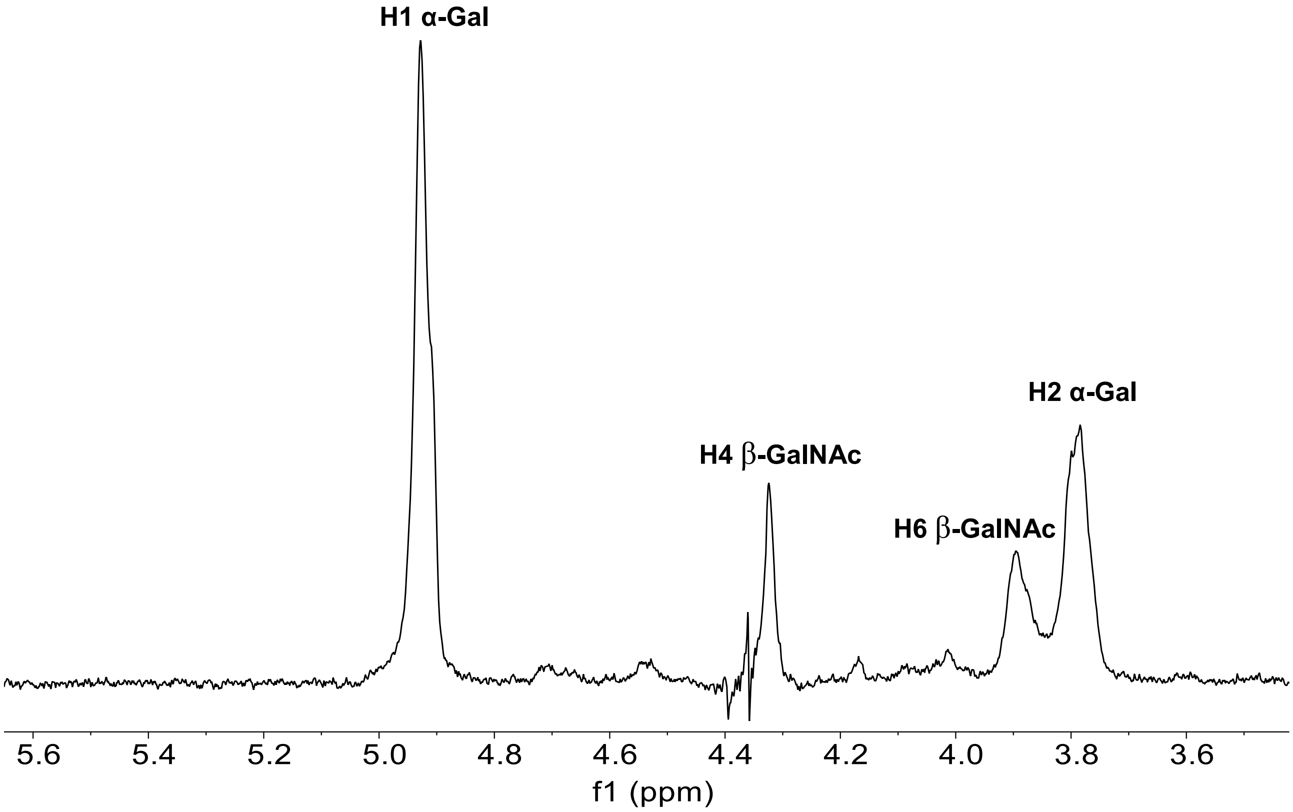

Supplementary Figure 11 | 1D NOESY spectrum obtained with selective excitation of H1 of the 6-linked α-Gal at 4.92 ppm present in the SL1 fraction. Inter-residues n.o.e. effects were detected between H1 of α-Gal and H4 and H6 of β-GalNAc, thus confirming the sequence α-Gal(1-4)β-GalNAc. Assignments are indicated.

a [→3)-β-D-Qui*p*NAc4NAc-(1→3)-β-D-Gal*p*NAc-(1→3)-α-D-Glc*p*NAc-(1]$_n$

4

↑

1

β-D-Gal*p*(3,4-*S*-Pyr)-(1→6)-β-D-Gal*p*(3,4-*S*-Pyr)-(1→6)-α-D-Gal*p*

b

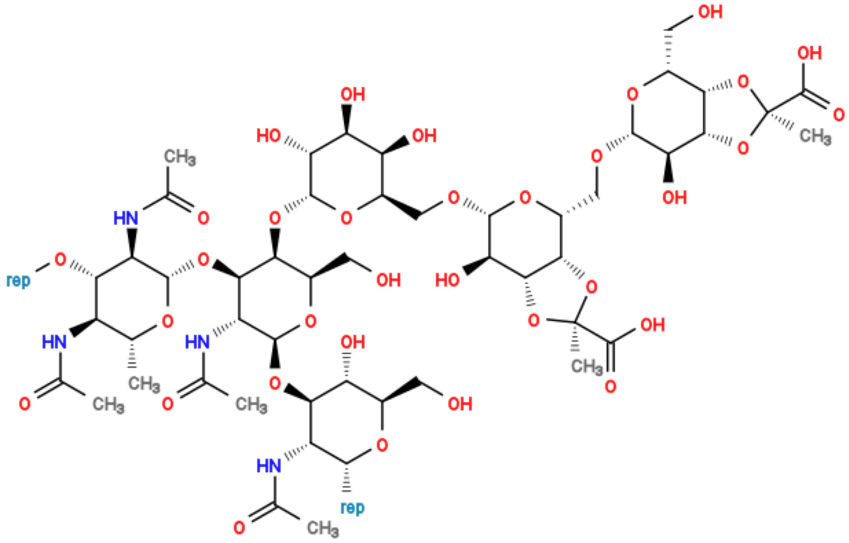

c

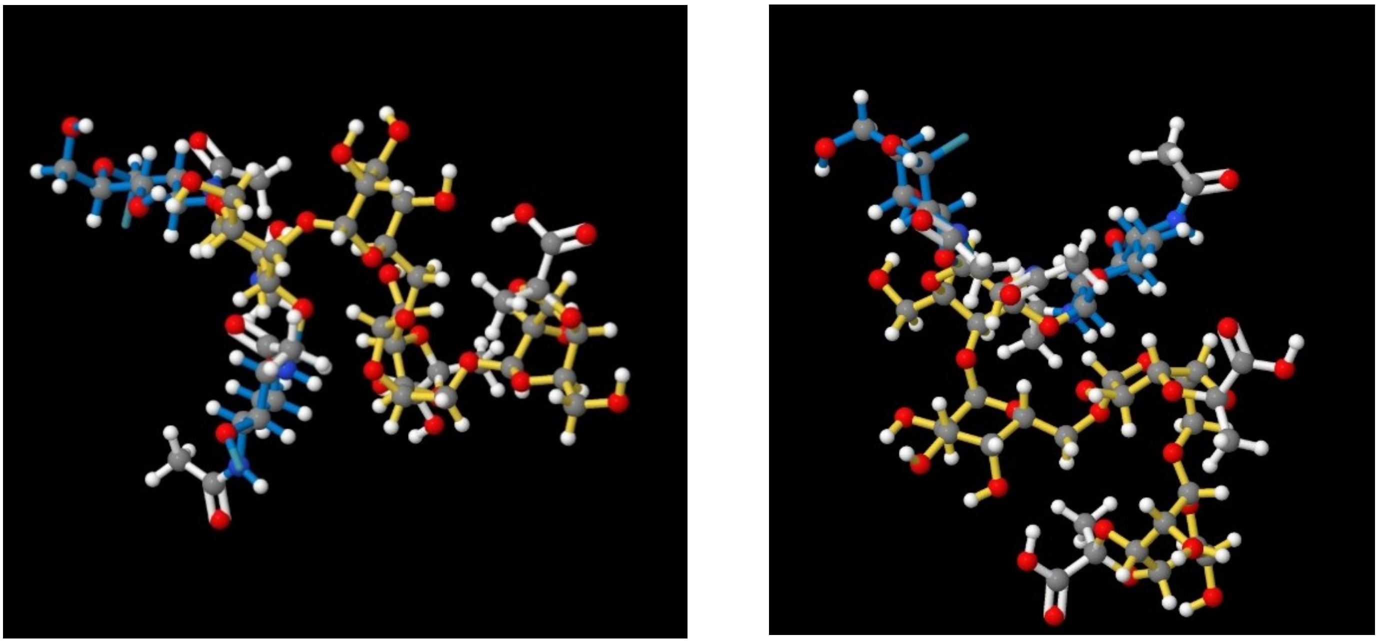

Supplementary Figure 12 | Repeating unit structure of EpsA-O. a structure of the EpsA-O repeating unit; **b** Haworth projection of the EpsA-O repeating unit; **c** two different views of the molecular structure of the repeating unit: blue bonds indicate Glc type rings; yellow bonds indicate Gal type rings. Structures b) and c) were obtained using the software available at http://csdb.glycoscience.ru/database/ (Scherbinina et al., 2021).

Supplementary Data 1

Functions assignments of the genes *epsE*, *epsH epsJ* and *epsF* in the *epsA-epsO* gene cluster of *B. subtilis*

The *epsA-epsO* gene cluster of *B. subtilis* 6051-HGW encodes 15 enzymes involved in the biosynthesis of the EpsA-O. The function of some of them has already been determined or predicted as follows:

- EpsA and EpsB are tyrosine kinases that regulate Eps production (Gerwig et al., 2014);
- EpsC, EpsM and EpsN are responsible for the biosynthesis of *N*,*N'*-diacetylbacillosamine (QuiNAc4NAc) (Kaundinya et al., 2018);
- EpsK is a putative membrane transporter similar to the Wzx protein of *E. coli* (Roux et al., 2015):
- EpsL binds UDP-QuiNAc4NAc to the lipid acceptor undecaprenol phosphate (UndP) (Arnaouteli et al., 2021);
- EpsD adds the N-acetylglucosamine residue (Arbour at al., 2023) to the QuiNAc4Nac-UndPP.

Among the remaining genes, *epsE*, *epsH* and *epsJ* encode for CaZy GT-2 enzymes with an inverting mechanism of action, whereas *epsF* encodes for a GT-4 enzyme, with a retaining mechanism. Therefore, EpsF is the only GT which can bind the α-D-Galactose. This hypothesis was corroborated by the homology between EpsF and *Yersinia enterocolitica* galactosyltransferase WbcN (Supplementary Table 1), which catalyzes exactly the same reaction (Pinta et al., 2010). Moreover, *in silico* analysis showed that EpsF and WbcN belong to the GT4_CapH-like family protein, characterized by common motives thought to be involved in the formation of an "ATP-binding pocket" important for catalytic activity (Supplementary Figure 13). Among the remaining GTs, EpsE, EpsH, EpsJ, one must be an N-acetylgalactosaminyltransferase, and two galactosyltransferases. All these enzymes belong to the CAZy (http://www.cazy.org/) GT-2 family, characterized by two distinct domains: the donor and the acceptor binding sites at the -NH_2 and -COOH domains, respectively (Ünligil and Rini, 2000). Comparison of their sequences evidenced high similarity between EpsH and EpsJ (mainly in the -NH_2 domain, whereas their -COOH domains were quite different), while EpsE showed low similarity with both GTs (Supplementary Figure 14). Moreover, EpsH and EpsJ -NH_2 domains showed homology with the same region of *B. anthracis* GtsE galactosyltransferase (Supplementary Table 1), thus suggesting that these enzymes are galactosyltransferases. Unfortunately, it was not possible to distinguish which enzyme catalyzes the addition of the first or the second galactose, because neither homologous -COOH domain sequences nor a known GT forming the linkage β-D-Gal*p*-(1→6)-β-D-Gal*p* or β-D-Gal*p*-(1→6)-α-D-Gal*p* was found in

available structural databases. By exclusion, EpsE was assigned to the N-acetylgalactosaminyltransferase that binds β-D-GalNAc*p* to C3 of α-D-GlcNAc*p*. Such hypothesis was also supported by its homology with the GalNAc*p* binding domain of *E. coli* K4 chondroitin polymerase (Supplementary Table 1) (Osawa et al., 2009). Finally, EpsG belongs to the GT-C superfamily, which are transmembrane proteins with 7 to 13 transmembrane domains exhibiting inverting glycosyltransferase activity starting from a polyisoprenol-linked donor substrate (Albuquerque et al., 2019). This finding, and the homology with the Wzy protein from *A. baumannii* (Kenyon et al., 2017) (Supplementary Table 1) suggest that EpsG is the polymerase that catalyzes the addition of RUs on the outer side of the membrane.

In the *epsA-epsO* cluster the genes responsible for the biosynthesis and activation of the common sugars Gal, GlcNAc and GalNAc are absent and they are probably located elsewhere, as it happens in other bacterial genomes.

Supplementary Table 1. Similarity level between Eps enzymes and proteins with known function obtained by BLAST alignment. Eps proteins: length and Accession numbers are indicated; Homologous proteins: name of the protein, bacterial species where it was described, their Accession Number and function are indicated.

	**Homologous protein**		**Similarity**				
	**Description**	**Enzymatic activity**	**E value**	**% Coverage**	**% Identities**	**% Positives**	**% Gaps**
**EpsE** 278 aa WP_003244557.1	Condroitin synthase *E. coli* 2Z87_A	GT-2 GalNAc*p* transferase (Osawa et al., 2009)	$3x10^{-16}$	54	28	45	4
**EpsF** 384 aa WP_003228257.1	WbcN *Y. enterocolitica* CAA87702	GT-4 Gal*p* transferase (Pinta et al., 2010)	$2x10^{-17}$	79	27	42	14
**EpsH** 344 aa WP_003243644.1	GtsE *B. anthracis* WP_000672693	GT-2 Gal*p* transferase (Chateau)	$1x10^{-12}$	45	33	48	5
**EpsJ** 344 aa WP_003228264.1			$5x10^{-8}$	43	32	51	4
**EpsI** 358 aa WP_003228262.1	WcuL *K. pneumoniae* BAT23553.1	Pyruvyl transferase (Pan et al., 2015)	$7x10^{-22}$	93	25	44	12
**EpsO** 322 aa WP_003228270.1			$4x10^{-77}$	98	39	59	7
**EpsG** 367 aa WP_003228259.1	Wzy *A. baumannii*	Sugar polymerase (Kenyon et al., 2017)	$7x10^{-18}$	56	31	49	15

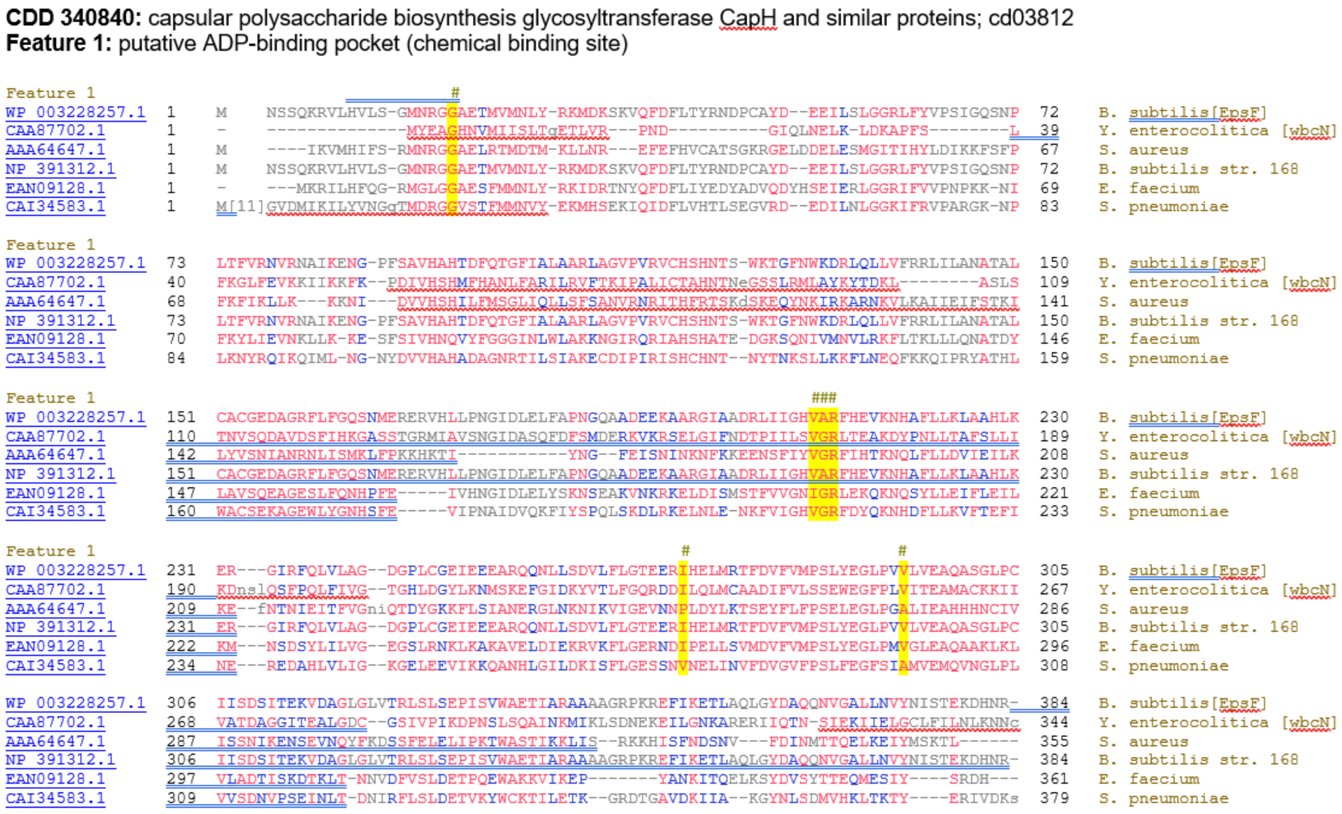

Supplementary Figure 13│COBALT Multiple alignment of EpsF of *B. subtilis* and its homologous wbcN of *Yersinia enterocolitica* with some representatives of the GT4-CapH-like Family Protein. The conserved feature residue patterns forming the putative ADP binding pocket, necessary for the catalytic activity of these enzymes, are highlighted (yellow and #).

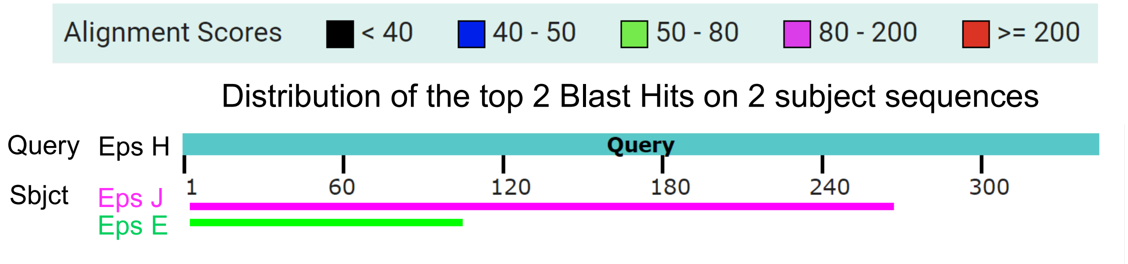

Supplementary Figure 14│Graphic Summary of BLAST alignments of EpsH with EpsJ and EpsE. Alignment of EpsH with EpsJ gave an E-value of $2x10^{-42}$, while alignment of EpsH with EpsE gave and E-value of $7x10^{-15}$.

```
NP 593959.1     1    M[30]DLQTLK[5]TSPSSSTSVDKKKPLFTKSPRNSASCESTITLQSNLLFTYYKHY[4]KKVALIGFPDHPNKGDSAI  109   S. pombe [Pvg1]
BAT23553.1      1    -    ------   ------------------------MHQNNLKNKLNEILPFIKDK  NEVIFLDYPVYLNVGDLLI  39    K. pneumoniae K30 [WclU]
WP 003228270.1  1    -    ------   --------MDSKHSMISLKQKLSGLLD-VIPKQS----------  -EIIYADYPLYGNVGDLFI  43    B. subtilis [EpsI]
WP 003228262.1  1    M    SLQSLK   INFAEWLLLKVKYP----SQYWLGAADQPVKAAAHQ--------  KKIILTLLPSHDNLGDHAI  58    B. subtilis [EpsO]

NP 593959.1     110  YVAEKKLLDALNIE--VVYITAQEADYSASELKSII[4]RDEFALAFHGGGNFGDLYPDHQHLRELVVRDFPSFTTISFP  188   S. pombe [Pvg1]
BAT23553.1      40   YHGTENFFKENNIN---VKLRRSNARYSVKEIEKYL   NPNTVILMQGGGNFGDLYPEFQKFRQDVVSSFPDTRIIILP  113   K. pneumoniae K30 [WclU]
WP 003228270.1  44   MKGTEAFFKEHGIR--VRKRWNPDNFPIGRKLDPNL   ----IIVCQGGGNFGDLYPYYQGFREKIVQTYPNHKIVILP  114   B. subtilis [EpsI]
WP 003228262.1  59   AYASKAFLEQEYPDfdIVEVDMKDIYKSAKSLIRSR   HPEDMVFIIGGGNMGDLYR-YEEWTRRFIIKTFHDYRVVQL  134   B. subtilis [EpsO]

NP 593959.1     189  QSVWYNEQQL    LEQASILYAENPNITLVTRDRQSYGFAVDAFGKhNEVLLTPDIVFFMGPIPEIREATPIThdvLILA  265   S. pombe [Pvg1]
BAT23553.1      114  QTAFFENEHE    LDKSVKAFGAHKDLIIFSRDTRSLSIFKKFTSQ---TFLMPDMAHSLWGTLPKSQKSKGT---LYLI  184   K. pneumoniae K30 [WclU]
WP 003228270.1  115  QSIYFQNKDN    LKRTAEIFSKHANLHIMTREKASYATAQAYFTT-NHIQLLPDMAHQLFPVIPTQQPSNQK---LRFI  187   B. subtilis [EpsI]
WP 003228262.1  135  PATAHFSDTK[5]LKRAQKIYNAHPGLLLMARDETTYQFMKQHFQE-KTILKQPDMVLYLDRSKAPAEREGVY-----MC  210   B. subtilis [EpsO]

NP 593959.1     266  RLDHEGGQQHGAEDYYRD    TLNAANLTYSVEDWLLWDPPV------AQNPDSSFDDRGQARYEAG---AEFLASARVV  333   S. pombe [Pvg1]
BAT23553.1      185  RNDKEINKVQRRLLEHKK[8]EDILTSKDLLMRKLCRKLDGIGGVTNLSVLKNISNVIWYNYTYKMVQRYALYFTSHEKV  269   K. pneumoniae K30 [WclU]
WP 003228270.1  188  RTDHEANQALQEHAEAES    YDWRTVLSASDRRTIAFLQTLNVLNKKAGNPLPIAYIWEKYSDYIVQKAIRFFSRYESV  264   B. subtilis [EpsI]
WP 003228262.1  211  LREDQESVLQEEQRNRVK    AALCEEFGEIKSFTTTIGRRVSRDTR------------EHELEAL---WSKLQSAEAV  271   B. subtilis [EpsO]

NP 593959.1     334  ITDRLHAHILSTLMGIPHIVVENSQmGKITNYHN   TWLHGCTLDGVSVVVDSVDKALSLLLEWNEAGYF       401   S. pombe [Pvg1]
BAT23553.1      270  VTSRMHGHIFSCLLSLPNDVIDNAY-GKNSGYFK   ---EWTYDIDGTKLLEE-----------------       316   K. pneumoniae K30 [WclU]
WP 003228270.1  265  ETSRLHGHILSSLLQKENTVIDNSY-GKNANYFH   TWMEGVPS---TRLIQHASKKENLPAHM------       322   B. subtilis [EpsI]
WP 003228262.1  272  VTDRLHGMIFCALTGTPCVVIRSFD-HKVMEGYQ[8]KLIEHPEPERVTAAVNELLTKETSRAGFPRDVYF[12]  358   B. subtilis [EpsO]
```

Supplementary Figure 15 | COBALT Multiple alignment of EpsI and EpsO pyruvyltransferases of *B. subtilis* with the enzymes WclU of *Klebsiella pneumoniae* and Pvg1 of *Saccharomyces pombe*. Both enzymes catalyze the pyruvylation of a galactose residue. In Pvg1 the binding sites for galactose (balck circles) and for pyruvate (black diamond) have been identified by crystallography (Higuchi et al., 2016)

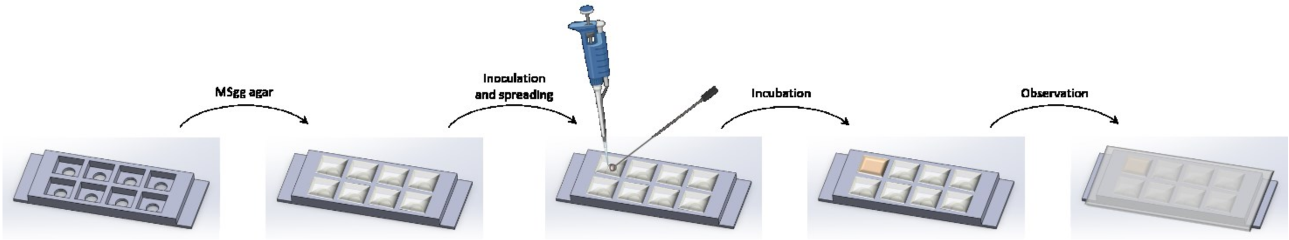

Supplementary Figure 16 | The workflow for preparing slides for observing biofilms under a microscope. The biofilm growth on solid MSgg agar was obtained in custom 3D printed 8-well chambered slides. The cover glass rested on the slide without touching the biofilm surface.

Supplementary References

Albuquerque-Wendt, A., Hütte, H. J., Buettner, F. F. R., Routier, F. H. & Bakker H. Membrane Topological Model of Glycosyltransferases of the GT-C Superfamily. *Int. J. Mol. Sci.* **20**, 4842; 10.3390/ijms20194842 (2019).

Arbour, C. A. et al. Defining Early Steps in *B. subtilis* Biofilm Biosynthesis. *mBio* 14:e00948-23. https://doi.org/10.1128/mbio.00948-23.

Arnaouteli, S., Bamford, N. C., Stanley-Wall, N. R. & Kovács, Á. T. *Bacillus subtilis* biofilm formation and social interactions. *Nat. Rev. Microbiol.* 19, 600-614 (2021). https://doi.org/10.1038/s41579-021-00540-9.

Chateau, A. et al. Distinct pathways carry out α and β galactosylation of secondary cell wall polysaccharide in *Bacillus anthracis*. *J. Bacteriol.* 202:e00191-20. (2020). https://doi.org/10.1128/JB.00191-20.

Gerwig, J., Kiley, T. B., Gunka, K., Stanley- Wall, N. & Stulke, J. The protein tyrosine kinases EpsB and PtkA differentially affect biofilm formation in *Bacillus subtilis*. *Microbiology* **160**, 682-691 (2014).

Higuchi, Y. et al. A rationally engineered yeast pyruvyltransferase Pvg1p introduces sialylation-like properties in neo-human-type complex oligosaccharide. *Sci. Rep.* **6**, 26349 (2016). https://doi.org/10.1038/srep26349

Kaundinya, C. R., Savithri, H. S., Rao, K. K. & Balaji, P. V. EpsM from *Bacillus subtilis* 168 has UDP-2,4,6-trideoxy-2-acetamido-4-amino glucose acetyltransferase activity in vitro. *Biochem. Biophys. Res. Commun.* **505**, 1057-1062 (2018).

Kenyon, J. J. et al. The KL24 gene cluster and a genomic island encoding a Wzy polymerase contribute genes needed for synthesis of the K24 capsular polysaccharide by the multiply antibiotic resistant *Acinetobacter baumannii* isolate RCH51. *Microbiology* **163**, 355-363 (2017) https://doi.org/10.1099/mic.0.000430.

Osawa, T. et al. Crystal structure of chondroitin polymerase from *Escherichia coli* K4. *Biochem. Biophys. Res. Comm.* **378**, 10-14 (2009). https://doi.org/10.1016/j.bbrc.2008.08.121

Pan, Y. J. et al. Genetic analysis of capsular polysaccharide synthesis gene clusters in 79 capsular types of *Klebsiella* spp. *Sci. Rep.* **5**, 15573; 10.1038/srep15573 (2015).

Pinta, E., et al. Characterization of the Six Glycosyltransferases Involved in the Biosynthesis of *Yersinia enterocolitica* Serotype O:3 Lipopolysaccharide Outer Core. *J. Biol. Chem.* **285**, 28333-28342 (2010) https://doi.org/10.1074/jbc.M110.111336.

Roux, D., et al. Identification of Poly-N-acetylglucosamine as a Major Polysaccharide Component of the *Bacillus subtilis* Biofilm Matrix. *J. Biol. Chem.* **290**, 19261-19272 (2015). https://doi.org/10.1074/jbc.M115.648709.

Scherbinina, S. I., Frank, M. & Toukach, P. V. Carbohydrate Structure Database oligosaccharide conformation tool. *Glycobiology*, 32, 460-468 (2022). https://doi.org/10.1093/glycob/cwac011.

Ünligil, U. M., & Rini, J. M. Glycosyltransferase structure and mechanism. *Curr. Opin. Struct. Biol.* **10**, 510-517 (2000). https://doi.org/10.1016/s0959-440x(00)00124-x.
